# Comparison of Methods for Analyzing Environmental Mixtures Effects on Survival Outcomes

**DOI:** 10.1007/s40572-025-00500-y

**Published:** 2025-11-01

**Authors:** Melanie N. Mayer, Arce Domingo-Relloso, Marianthi-Anna Kioumourtzoglou, Ana Navas-Acien, Brent A. Coull, Linda Valeri

**Affiliations:** 1 Division of Pulmonary and Critical Care Medicine, Department of Medicine, Perelman School of Medicine, University of Pennsylvania, Philadelphia, PA USA; 2 Department of Biostatistics, Columbia Mailman School of Public Health, New York, NY USA; 3 Department of Environmental Health Sciences, Columbia Mailman School of Public Health, New York, NY USA; 4 Department of Biostatistics, Harvard T.H. Chan School of Public Health, Boston, MA USA; 5 Department of Epidemiology, Harvard T.H. Chan School of Public Health, Boston, MA USA; 6 Department of Epidemiology, Brown University School of Public Health, Providence, RI USA; 7 Institute at Brown University for Environment and Society, Brown University, Providence, RI USA

**Keywords:** Environmental mixtures, Survival analysis, Machine learning, Continuous exposures

## Abstract

**Purpose of Review:**

Estimating the effect of environmental mixtures on survival outcomes is common in epidemiological studies, yet the applicability and performance of advanced mixture modeling methods in this context remains underexplored. In this review, we identify available methods for this context and evaluate their performance via simulations.

**Recent Findings:**

We compared five methods – Cox Proportional Hazards (with/without penalized splines), Cox Elastic Net, Bayesian Additive Regression Trees (BART), and Multivariate Adaptive Regression Splines (MARS). Simulations showed log-linear models achieved low coverage when estimating individual exposure and mixture effects, especially under high exposure correlations and proportional hazards violations. More flexible models exhibited higher variability but improved coverage in effect estimation.

**Summary:**

While flexible models were better able to estimate mixture effect on survival outcomes compared to more constrained models for most simulation scenarios, they still introduced bias and often had high variability. Given real-world constraints like limited sample sizes and high censoring, there likely remains significant complexities for the application of flexible modeling for environmental mixtures for the survival analysis contexts. We recommend evaluating if findings are consistent across methods.

**Supplementary Information:**

The online version contains supplementary material available at 10.1007/s40572-025-00500-y.

## Introduction

Environmental mixtures studies often consist of multiple continuous and correlated exposures with complex relationships, such as nonlinearity and interactions, on an outcome of interest [1, 2]. Historically, it has been common to evaluate the effect of a single exposure at a time; however, there has been a recent shift to consider the complete mixture to better capture what occurs in nature [2–4]. Moreover, environmental epidemiological studies are often concerned with the impact of exposures on the time of development of a particular outcome, such as death or disease, which require specialized modeling approaches [5–7]. Despite growing interest in mixture modeling, most evaluations have focused on continuous or binary outcomes [8–11]. The application of mixture models to survival outcomes remains understudied and key survival-specific challenges such as the choice of estimand for measuring a mixture effect, the adaptation of modeling methods, and accounting for the timing and censoring of the event requires additional attention.

We introduce a practical framework for evaluating the joint effect of environmental exposures on a survival outcome. We demonstrate how to quantify the mixture effect using both the hazard ratio and the survival probability difference [12]. We also visualize individual exposure-response functions by estimating survival probabilities across a range of concentrations of an individual exposure, holding all other exposures constant. We compare a range of traditional and modern statistical methods, specifically, Cox Proportional Hazards (PH)-based approaches and more flexible, machine learning (ML) style approaches, selected for their widespread use in environmental epidemiology and their potential to capture complex exposure-response relationships, respectively. The methods evaluated include Bayesian Additive Regression Trees [13], Cox Elastic Net [14], Cox Proportional Hazards Model [15], Cox Proportional Hazards Model with penalized splines [16], and Multivariate Adaptive Regression Splines [17]. We offer insight into their applications and limitations, provide an open-access vignette illustrating how to implement these methods in R, and conduct a simulation study to evaluate their performance under various real-world scenarios, including complex exposure-outcome relationships, high dimensional exposure sets, highly correlated exposures, and proportional hazards assumption violations. To illustrate how our proposed framework can be applied in practice, we include a real-world data application estimating the joint effect of a six-metal mixture on incident cardiovascular disease in the Strong Heart Study (SHS), a cohort study of American Indian adults, in the Supplemental Material [18].

This work provides methodological insight and practical guidance for evaluating environmental mixtures in the context of survival outcomes. By offering insights into these methods we aim to guide future studies and support ongoing methodological development for analyzing environmental mixtures in the context of survival outcomes.

## Methods

### Quantities of Interest

We consider the scenario where we have baseline measures on multiple environmental exposures and confounders. Subjects are followed through a follow-up period where the occurrence of the event of interest is monitored. While our primary focus is on the overall effect of the environmental mixture on survival outcomes, we also estimate individual and pairwise interaction effects to underscore how varying research questions can influence model performance and study findings, and to further provide practical guidance, as these effects are more commonly emphasized in existing literature. To quantify each of these three types of effects, we consider popular point estimates for time-to-event outcomes. Traditionally, the Cox PH model, which naturally estimates hazard ratios (HRs), has been used to model survival outcomes [15]. Thus, environmental epidemiologists commonly used HRs to quantify exposure effects. To be more comprehensive, we also consider effects with respect to the survival probability difference (SPD). Different estimands may be of interest for public health decision-making, and it has been shown that conclusions can vary depending on the choice of scale being used (i.e. ratio vs. difference measures) [19]. Furthermore, when it comes to survival analysis, the clinical meaning of a HR can be difficult to interpret, especially when the underlying PH assumption incorporated into the Cox PH model is violated. In certain circumstances, a model-free estimand with clearer clinical and analytic interpretability, such as the SPD, may be preferred [20].

To estimate the effect of the overall environmental mixture $\bf M$, composed of $\:J$ components, on a survival outcome, we use $\:{\lambda\:}^q\left(t\right|\mathbf{C})$ and $\:S^q\left(t\right|\mathbf{C})$ to denote the hazard and survival probability, respectively, at time $\:T=t$, when exposed to mixture components each at their respective $\:{q}^{th}$ percentiles, conditional on all confounders $\bf C$. We quantify the effect of an environmental mixture on the survival outcome as the HR and the SPD at time $\:t$ for an interquartile range (IQR) change in each exposure, denoted as $\:{\lambda\:}^{75}\left(t\vert \mathbf{C}\right)/{\lambda\:}^{25}\left(t\right|\mathbf{C})$ and $\:S^{75}\left(t\vert \mathbf{C}\right)-S^{25}\left(t\right|\mathbf{C})$, respectively. That is, we consider the HR and SPD comparing the hazards and the survival probabilities when each exposure $\:{M}_{1},\:\:{M}_{2},\dots\:,{M}_{J}$ is set to their 75th percentile compared to their 25th percentile. While comparing all exposures shifting jointly from their 25th to 75th percentiles may not be fully observable in practice, we considered an IQR contrast because it is a commonly used, standardized metric that reflects lower and higher exposure concentrations observed in the population. However, the choice of mixture contrast can differ depending on context-specific considerations and is easily modifiable in practice. As seen in their mathematical formulations, the HR and SPD depend on time. Thus, to estimate these point estimates, a time, $\:{t}_{spec}$, must be specified. This choice can be motivated by contextual relevance, such as the median survival time or a clinically meaningful follow-up period. 

Our quantification of individual exposure and pairwise interaction effects, along with the mathematical formulations of these quantities and the estimands described above, are presented in Supplemental Material S1. These quantities, selected for their frequent use or interpretability, are not exhaustive. Other quantities may also be of interest to researchers. For example, environmental epidemiologists may be interested in exposure-response functions, especially in the presence of nonlinear effects, where a contrast, such as the IQR, may not fully capture the complexity of the relationship. Thus, in addition to estimating the overall mixture effect, we also consider each method’s ability to estimate the survival probability as a function of an individual exposure’s concentration at $\:{t}_{spec}$, holding all other exposures and covariates at their median values. This provides a visualization of how the models estimate the relationships between an exposure and the outcome and, in practice, provides additional insight into how individual exposures contribute to the mixture effect.

### Methods for Survival Analysis

We aimed to identify advanced, readily available modeling methods for survival analysis with an environmental mixture exposure. We prioritized methods that: (1) support survival outcomes; (2) support continuous exposures; (3) allow for potential nonlinear effects and interactions among the exposures on the outcome; (4) allow for inference (i.e., provides estimates and confidence intervals/standard errors); and (5) are implementable in the statistical computing software R. Additionally, we preferred methods that perform variable selection and flexibly estimate exposure-response relationships without specifying their functional form. We compared traditional methods used in environmental mixture studies – Cox Proportional Hazards (PH) model with and without penalized splines and Cox Elastic Net (EN) – and two ML methods - Bayesian Additive Regression Trees (BART) and Multivariate Adaptive Regression Splines (MARS) [13–17]. Other methods, such as Principal Component Analysis, Weighted Quantile Sum Regression, Gaussian Process Regression, Bayesian Kernel Machine Regression, and Random Survival Forests, were considered but excluded due to limitations such as lack of inference or excessive computational burden [21–26]. The selected methods can be categorized into two modeling frameworks: those based on the Cox PH model that require the PH assumption, and those that use a discrete time approach and allow effects to vary across time segments [27]. Each method is numbered and described below, with Table 1 summarizing their relevant characteristics. For more details on the methods, see Supplemental Material S2, which includes Table S2 summarizing the hyperparameters for each method, as well as the fixed values or cross-validation grids used for each.

**Table 1:** Summary of characteristics of selected modeling methods

	Time-to-event outcome	Multiple continuous exposures	Non-linear and interaction effects on the outcome	Inferences (point estimates and confidence intervals)	Variable selection	Automated functional form of covariates	No Proportional Hazard Assumption
Cox Proportional Hazards Model (Cox PH)	✓	✓		✓			
Cox Proportional Hazards Model with Penalized Splines (Cox PH-ps)	✓	✓	✓	✓			
Cox Proportional Hazards Model with Elastic Net (Cox EN)	✓	✓		✓	✓		
Multivariate Adaptive Regression Splines (MARS)	✓	✓	✓	✓	✓	✓	✓
Bayesian Additive Regression Trees (BART)	✓	✓	✓	✓	✓	✓	✓

#### Modeling Framework 1 - Proportional hazards models

This approach models the hazard function as $$\:\lambda\:\left(t;\bf{m},\bf{c}\right)={\lambda\:}_0\left(t\right)e^{f(\mathbf{m},\bf{c})}$$. This implies that while $$\:{\lambda\:}_{0}\left(t\right)$$ can vary over time, the hazard of the event given a set $$\:\{\mathbf{m},\mathbf{c}\}$$ is a constant multiple of the hazard for another set $$\:\{\mathbf{m}^{\ast},\mathbf{c}^{\ast}\}$$, where $$\:\{\mathbf{m},\mathbf{c}\}$$$$\:\neq\:\:\{\bf{m}^{\ast},\bf{c}^{\ast}\}$$, implicitly imposing the PH assumption. The following methods follow this general form but have different approaches for estimating $$\:f\left(\bf{m},\bf{c}\right)$$..

1. *Traditional Cox Proportional Hazards (Cox PH).* For the Cox PH model, the function $\:f(\mathbf{m},\mathbf{c})$ in the exponential portion of the hazards is modeled as a linear function of the $\:J$ exposures and $\:L$ confounders, implying log-linear effects. Although among the most common models used, it also among the most restrictive. To estimate the coefficients of this model, we used R package *survival* (version 3.2-7).
2. *Traditional Cox Proportional Hazards with Interactions (Cox PH w. Int).* We consider the Cox PH model with all possible two-way interactions between the exposures.
3. *Cox Proportional Hazards with Penalized Splines (Cox PH-ps)*. This extension of the traditional Cox PH model relaxes the log-linearity assumption by allowing one to use penalized splines [28]. We applied penalized splines to all exposures and, where feasible, their two-way interactions, while confounders were entered linearly. Although this approach improves flexibility, it requires pre-specifying which terms are modeled nonlinearly and can be less computationally and statistically efficient due to its reduced parsimony compared to the traditional Cox PH model. Smooth functions were estimated using the R package *mgcv* (version 1.8–33).
4. *Cox Elastic Net (Cox EN).* This model assumes the same form for the hazard function as the traditional Cox PH model, that is it is also log-linear, but estimates the coefficients differently. EN imposes constraints that shrink exposure-related coefficients toward zero, reducing overfitting and enabling variable selection. EN has been found to be particularly advantageous for high-dimensional, correlated exposure sets where some components might be redundant. In our implementation, confounder coefficients were excluded from penalization to ensure they were not subject to shrinkage. The coefficient estimation procedure, including its associated hyperparameters related to L1/L2 penalty mixing and regularization strength, is described further in Supplemental Material S2. Estimation was performed using the R package *glmnet* (version 4.1-3).
5. *Cox Elastic Net with Interactions (Cox EN w. Int).* We consider the Cox EN including all possible two-way interactions between the exposures, with both main effects and interaction terms subject to penalization.

#### Modeling Framework 2 - Discrete-time survival models

This approach relaxes the PH assumption and enables survival predictions using binary classification methods [27]. Accordingly, we used a discrete-time survival analysis framework to apply flexible models not available for survival outcomes in R. Briefly, consider each subject’s corresponding triple $\it \:(t_i,{\delta\:}_i,\mathbf{x}_i)$. For subject $\:i=1,\dots\:,\:n$, $\:{t}_{i}$ denotes the observed time to event or censoring, $\:{\delta\:}_{i}$ denotes the indicator of whether the event occurred or was censored, and $\it \:\mathbf{x}_i$ denotes the joint set of observed exposures and confounders. Time is discretized into $\:R$ bins of event/censoring times delineated by its corresponding $\:1/R,2/R,\dots\:,1$ quantiles. An augmented dataset is then created such that a subject has multiple corresponding observations for each distinct discretized time up to the bin including their observed $\:{t}_{i}$. An observation now refers to a subject-time, where confounders/exposures are repeated for the number of rows corresponding to each subject in the new augmented dataset. A vector of a binary variable, $\it \:\mathbf{Y} _i$, is created to indicate event status per subject-discretized time. For example, consider time discretized into $\:R=3$ bins: [0,1), [1,2), and [2,3). If subject $\:i$ experienced the event at $\:{t}_{i}=1.5$, they will have two observations in the augmented dataset and $\:\mathbf{Y}_i = \left(0,1\right)$. Conversely, had this subject been censored at this time, they would also have two rows pertaining to them in the augmented dataset, however $\:\mathbf{Y}_i=\left(0,0\right)$. For more detail and an example see Sparapani et al. [13]. 

$\:\mathbf{Y}$ can now be treated as the outcome of interest and one can apply methods for binary outcomes to the augmented dataset with the discretized time included as a covariate. This is modeled as $\:g\left(\mu\:\right)=f(\mathbf{m},\mathbf{c},t)$, where $\:g(.)$ is a monotonic link function, $\:\mu\:=E\left(Y\right|\mathbf{m},\mathbf{c},t)$ is the expected probability of experiencing the event, and $\:f(\mathbf{m},\mathbf{c},t)$ is a function of the exposures, confounders and time estimated through an algorithm. The quantities of interest can be estimated from the discrete time approach for survival outcomes using known formulas shown in Supplemental Material S2. The following algorithms have different approaches for estimating this $\:f(\mathbf{m},\mathbf{c},t)$..

6. *Multivariate Adaptive Regression Splines (MARS).* The MARS algorithm creates a piecewise linear model convenient for modeling nonlinear and interaction relationships of the exposures while still maintaining some interpretability [29]. Nonlinear relationships can be captured by binning the range of values for each exposure into smaller sections, split by values referred to as knots, and creating linear regression models for each section. The relationship of an exposure with the outcome thus differs over different ranges of the exposure concentrations. The relationship between the exposures and time with the outcome are modeled by summing over multiple piecewise linear functions as well as products of piecewise linear functions to model interactions. MARS can be computationally intensive, and a large sample size is needed to consider a model with a high degree of interaction. For our MARS model adaptation to discrete time survival analysis, we used the logit link function. We used the R package *earth* (version 5.3.1) to fit the models and *caret* (version 6.0–90) to cross-validate for the hyperparameters pertaining to the number of terms retained and the degree of interaction.
7. *Bayesian Additive Regression Tree (BART).* BART is a nonparametric Bayesian regression method which approximates $\:f(\mathbf{m},\mathbf{c},t)$ using a sum of trees approach [30]. A Bayesian approach is used to fit the trees, with a prior imposed to regularize the fit by keeping individual tree effects small. Interactions and nonlinearities are naturally incorporated into the tree structure. By imposing a Bayesian framework, credible intervals are also easily produced, easing one’s ability to measure uncertainty. To implement BART for the discrete-time survival setting, we used a logit link function and fit the model using the R package BART (version 2.9.0) with default priors.

### Simulation Study

To assess the performance of each method, we simulated data to replicate four real-world scenarios. For each scenario, we simulated $\:F=500$ datasets with $\:n=3000$ observations each. Approximately 67% of the observations were censored. For details on how the simulated datasets were produced, see Supplemental Material S3. The scenarios considered include:

- *Scenario 1 – Base-Case Scenario*: The mixture consists of 5 components with low to moderate correlations across exposures and nonlinear effects on the time-to-event outcome. The PH assumption holds.
- *Scenario 2 – High Dimensional Scenario*: Same as Scenario 1, but with 10 mixture components.
- *Scenario 3 – High Correlation Scenario*: Same as Scenario 1, but with higher correlations across mixture components.
- *Scenario 4 – Non-proportional Hazards Scenario*: Same as Scenario 1, but with violations of the PH assumption.

We estimated our quantities of interest for all simulated datasets. For the discrete-time survival analysis models, we used $\:R=5$ discretized time bins to balance the granularity of follow-up time with computational feasibility. To assess the sensitivity of this choice, we compared model performance when $\:R=5$ versus $\:R=10$ for Scenario 1. For illustrative purposes, we used the 80th percentile of observed follow-up time as our pre-specified time of interest ($\:{t}_{spec})$. To evaluate the impact of this selection, we also examined model performance at an alternative time point, $\:{t}_{spec}=10$, in Scenario 1. In addition, we explored model performance sensitivity to censoring, a topic highly relevant in survival analysis.

Methods’ performances for the simulation study were summarized in terms of their bias and efficiency in estimating the true values of the quantities of interest described. The relative bias for each method was defined as $\:({{\Omega\:}}_{truth}-{\widehat{{\Omega\:}}}_{f}\:)/{{\Omega\:}}_{truth}$, where $\:{{\Omega\:}}_{truth}$ is the true value and $\:{\widehat{{\Omega\:}}}_{f}$ is the estimated value for the quantity of interest based on the $\:f=1,\dots\:,F$ simulated dataset. The standard deviation (SD) for a given simulated estimate was estimated either by taking the SD of the estimates from the bootstrap samples or, in the case of the Bayesian methods, the SD of the posterior distribution of the quantity of interest. Coverage probability was computed as the percentage of the confidence intervals estimated that included the true value, $\:(1/F){{\Sigma\:}}_{f=1}^{\text{F}}\:I\{{\widehat{{\Omega\:}}}_{f,ll}\le\:{{\Omega\:}}_{truth}\le\:\:{\widehat{{\Omega\:}}}_{f,ul}\}$ where $\:{\widehat{{\Omega\:}}}_{f,ll}$ and $\:{\widehat{{\Omega\:}}}_{f,ul}$ refer to the estimated lower and upper limits of the confidence/credible intervals using the $\:f$ simulated dataset. To quantify the method’s performance in estimating the exposure-response curve, we used the mean integrated squared error (MISE):

$$\begin{aligned} \:MISE\:=\frac{1}{F}{{\Sigma\:}}_{f=1}^{\text{F}}\frac{1}{\left|\right\{0.05,\:0.10,\:\dots\:,\:0.95\left\}\right|}\\{{\Sigma\:}}_{q=\:0.05,\:0.10,\:...,\:0.95}{\left({\widehat{S}}_{j,f}^{q}\left(t|{{\mathbf{M}}}_{-j},\mathbf{C}\right)-\:{S}_{j}^{q}\left(t|{{\mathbf{M}}}_{-j},\mathbf{C}\right)\right)}^{2} \end{aligned}$$

$\:\widehat S_{j,f}^q\left(t\vert \mathbf{M}_{-j},\mathbf{C}\right)$ refers to the estimated survival probability at time $\:t$ using the $\:{f}^{th}$ simulated dataset when exposed to environmental mixture component $\:j$ at it’s $\:{q}^{th}$ percentiles, conditional on all other exposures $\:\mathbf{M}_{-j}$ and all confounders $\:\mathbf{C}$. 

To further demonstrate the applicability of the proposed methods to a real-world scenario of multiple, continuous exposures with a survival outcome, we conducted a mixture analysis using prospective cohort data from the SHS. We estimated the joint effect of a six-metal mixture on incident cardiovascular disease, applying the same modeling methods compared in the simulation study. Full methodological details are provided in Supplemental Material S5.

## Results

We evaluated the performance of the seven models in estimating the effect of an environmental mixture on a survival outcome across four simulation scenarios. Findings from sensitivity analyses evaluating the number of time bins, choice of time point, and presence of censoring are shown in Supplemental Material S4. Additionally, results from SHS application are presented in Supplemental Material S5, demonstrating the practical implementation of our proposed framework and providing real-world context for the differences in method performance observed in the simulation study.

**Fig. 1:**
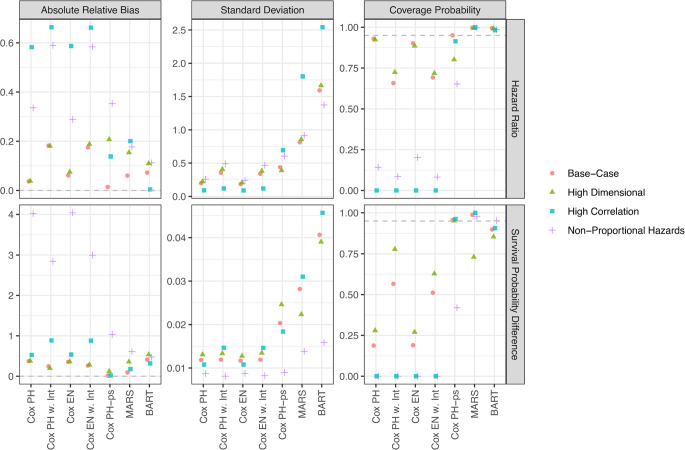
Performance of modeling methods in estimating the hazard ratio and the survival probability difference for an interquartile range change in all components of an environmental mixture. Results are shown across four simulation scenarios, indicated by point color and shape. Three performance metrics are shown: (1) absolute median relative bias, (2) median standard deviation, and (3) coverage probability (i.e., proportion of 95% confidence intervals that include the true effect). Each metric is calculated from 500 simulated datasets per scenario. Gray dashed horizontal lines indicate optimal values: 0 for absolute relative bias and 95% for coverage probability

Figure 1 compares model performance in estimating the HR and SPD from an IQR change in the mixture across four simulation scenarios. The 80th percentile of time used as $\:{t}_{spec}$ was 18.5, 18.5, 18.4, and 18.6 years for each of the four scenarios, respectively. In the Base-Case Scenario, the Cox PH-ps model exhibited the lowest absolute average relative bias for both HR (0.01) and SPD (0.01), while Cox PH w. Int and BART had the highest for HR (0.18) and SPD (0.41), respectively. Log-linear models (Cox PH and Cox EN, with and without interactions) generally had lower SDs than the more flexible models (Cox PH-ps, MARS, and BART), but at the cost of lower coverage probabilities. Only Cox PH-ps and MARS achieved over 95% coverage for both quantities. In the High Dimensional Scenario (10-component mixture), log-linear models performed similarly to the 5-component mixture scenario, while the flexible models showed varying degrees of increasing bias. Here, the absolute relative bias ranged from 0.04 (Cox PH) to 0.21 (Cox PH-ps) for HR, and 0.12 (Cox PH-ps) to 0.54 (BART) for SPD. No method achieved over 95% coverage for both quantities, and the coverage advantage of flexible models over log-linear models became slightly less consistent. For the High Correlation and Non-Proportional Hazards Scenarios, log-linear models performed worse, exhibiting higher absolute biases and lower coverage compared to flexible methods for both quantities. In the High Correlation Scenario, bias from log-linear models ranged from 0.58 to 0.66 for HR and 0.53 to 0.89 for SPD (from Cox PH to Cox PH w. Int, respectively) and resulted in zero coverage. In contrast, flexible models maintained comparable biases to other scenarios (HR: 0.00–0.20; SPD: 0.02–0.32) and achieved > 90% coverage, underscoring their relative robustness under high correlation. Similar results were seen in the Non-Proportional Hazards Scenario, except for Cox PH-ps, which also showed increased bias and reduced coverage under this assumption violation.

**Fig. 2:**
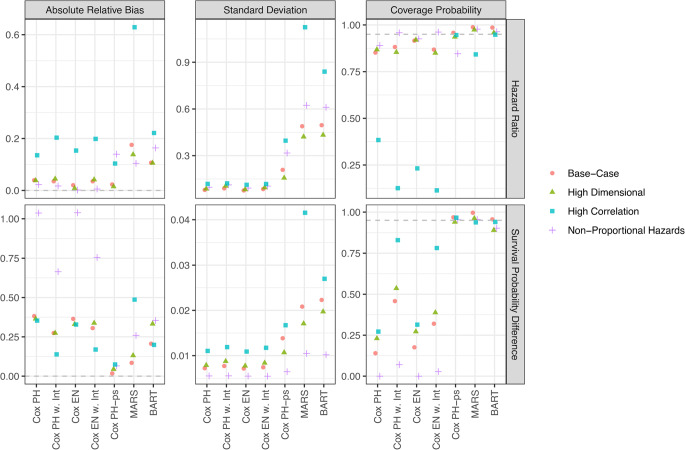
Performance of modeling methods in estimating the hazard ratio and the survival probability difference for an interquartile range change in a single exposure component within an environmental mixture. Results are shown across four simulation scenarios, indicated by point color and shape. Three performance metrics are shown: (1) absolute median relative bias, (2) median standard deviation, and (3) coverage probability (i.e., proportion of 95% confidence intervals that include the true effect). Each metric is calculated from 500 simulated datasets per scenario. Gray dashed horizontal lines indicate optimal values: 0 for absolute relative bias and 95% for coverage probability

Similarly, Fig. 2 compares model performance for estimating an IQR change in an individual exposure. These results show larger performance variation between HR and SPD estimands. For SPD estimation, model performance more closely resembles that seen for the mixture effect, such as the large drop in coverage for log-linear models, none of which achieve 95% coverage across scenarios. Although performance varies among flexible models across scenarios, Cox PH-ps consistently achieves the lowest bias while maintaining good coverage. Conversely, model performance for HR estimation differs from that of the mixture. Except for the High Correlation Scenario, log-linear models maintain low absolute relative bias and high coverage. These advantages diminish under higher correlation, where coverage for log-linear models drops sharply. Flexible models generally retain comparable performance across scenarios, except for a relative decline observed for Cox PH-ps under the Non-Proportional Hazards scenario and for MARS under the High Correlation scenario. MARS, which performed relatively well for mixture estimation, displays higher relative bias compared to all the other models for most scenarios.

**Fig. 3:**
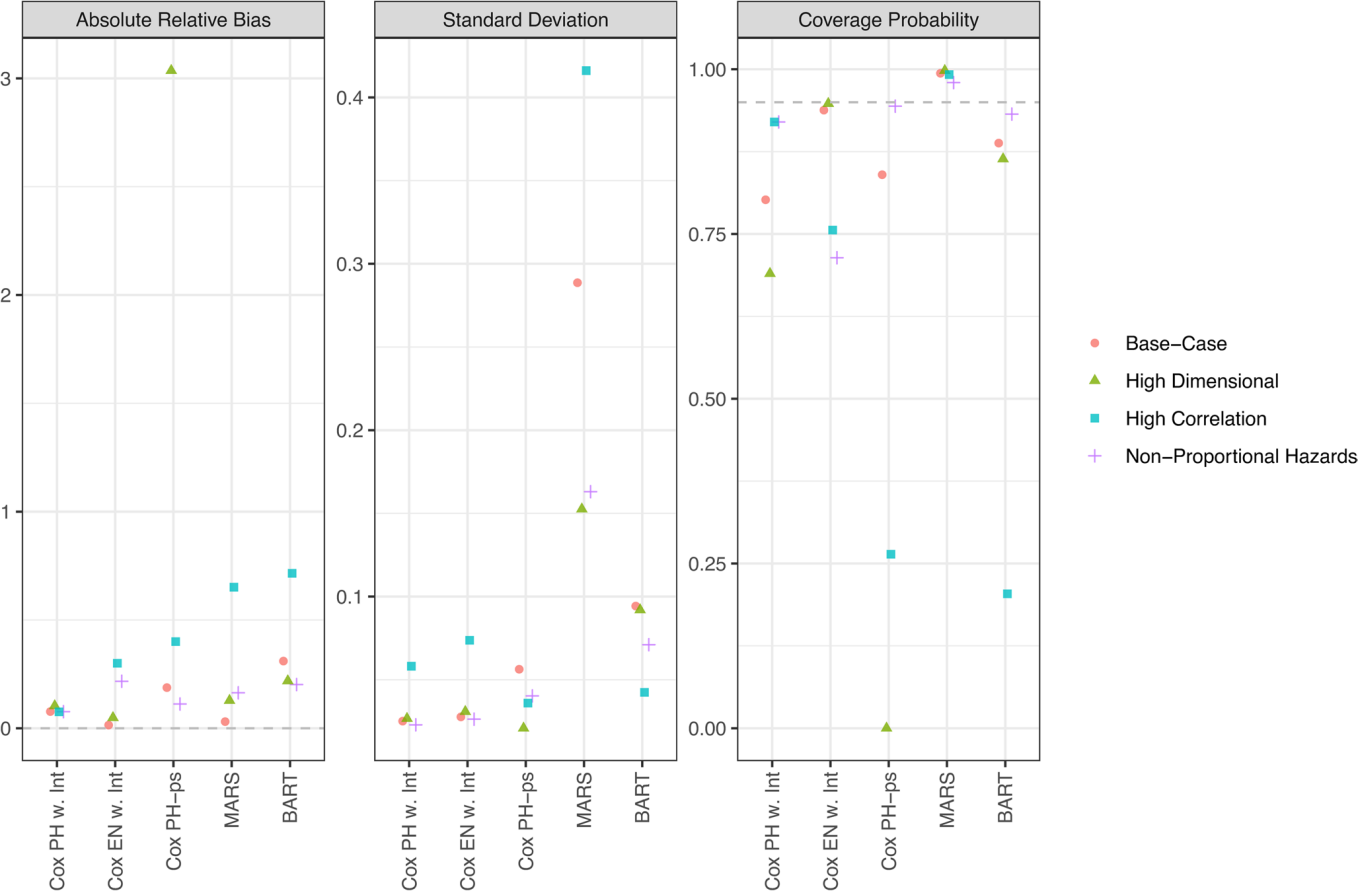
Performance of modeling methods in estimating the multiplicative interaction between two exposure components within an environmental mixture. Results are shown across four simulation scenarios, indicated by point color and shape. Three performance metrics are shown: (1) absolute median relative bias, (2) median standard deviation, and (3) coverage probability (i.e., proportion of 95% confidence intervals that include the true effect). Each metric is calculated from 500 simulated datasets per scenario. Gray dashed horizontal lines indicate optimal values: 0 for absolute relative bias and 95% for coverage probability

Figure 3 presents the performance of the modeling methods in estimating a multiplicative interaction. Cox PH and Cox EN do not include interaction terms between exposures and were therefore excluded. In the High Dimensional Scenario, Cox PH-ps also omitted interactions due to converge failure when including all two-way interactions among the ten exposure components, resulting in substantially increased bias. Overall, Cox PH w. Int maintained the most consistently low bias, while MARS achieved over 95% coverage, across all scenarios. Estimating interaction effects proved particularly challenging for most models in the High Correlation Scenario. For interaction effect estimation, it is preferable to use a modeling method that automatically allows for interactions. Among the methods considered, only MARS and BART naturally accommodate interactions without requiring explicit specification (assuming that MARS is tuned to include interactions of degree two or higher). Of these, MARS outperformed BART by achieving consistently lower bias and higher coverage, suggesting it as a promising choice when interaction effects are the primary focus.

**Fig. 4:**
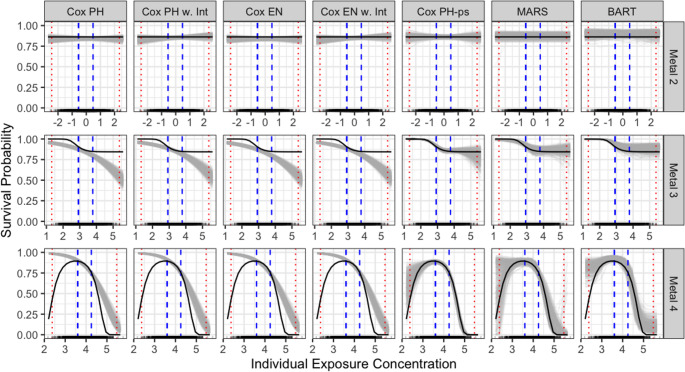
Performance of modeling methods in estimating survival probability by individual metal concentration, for three metals in the base-case simulation scenario; black curves indicate true curves, gray curves represent estimated curves, rug plots show density of metal from a randomly selected dataset, vertical blue dashed lines mark the 25th and 75th percentiles (IQR range), and red dotted lines mark the 5th and 95th percentiles (majority data range)

Figure 4 shows the estimated exposure-response curves for three individual exposures, estimated for each of the 500 simulated datasets under the Base-Case Scenario. The MISE for a single exposure across the different scenarios are provided in Supplemental Material S4 (Figures S5). Cox PH-ps, MARS, and BART performed well, estimating curves more closely aligned with the true curve (shown in black) across regions of denser exposure concentrations. However, their accuracy diminished at the extremes of the exposure range. Consistent with their performance for the point estimates, these models had substantial variability in their curve estimates. In contrast, the other methods provided more stable curve estimates but lacked flexibility to adequately capture non-linear relationships. Metal 2 was simulated such that it had no effect on the outcome, thus the true exposure-response curve is flat. All methods yielded low MISE values for metal 2, ranging from 0.0004 (Cox PH-ps) to 0.0026 (BART), regardless of their capability to perform variable selection. Metals 3 and 4, which were simulated with non-linear relationships on the outcome, had lower MISE for flexible models compared to log-linear models. For Metal 3, flexible methods had MISE values from 0.0007 to 0.0019, while log-linear methods ranged from 0.0124 to 0.0158. For Metal 4, flexible methods’ MISE ranged from 0.0053 to 0.0173, versus from 0.0478 to 0.0505 for log-linear models. For all three metals, Cox PH-ps had the lowest MISE.

## Discussion

We present a framework for estimating the effects of an overall mixture on a time-to-event outcome, shedding light on survival-specific considerations, such as the choice of estimand and statistical modeling methods. To facilitate the application of our approach, we provide an illustrative vignette demonstrating how to implement these methods in R (https://melanie-mayer.com/mixture_survival_vignette.html). Through simulations, we demonstrate that while no single method consistently outperforms others across all scenarios and estimands, flexible models are often advantageous, particularly in challenging settings such as high exposure correlations or violations of the proportional hazards assumption. Although our primary focus was on simulation-based evaluation, our supplemental application to the SHS illustrates the potential of this framework to yield interpretable estimates in complex real-world exposure settings.

Simulation results highlighted key trade-offs between flexible and constrained modeling approaches. Flexible methods (Cox PH-ps, MARS, BART) generally provided improved coverage compared to constrained methods (Cox PH, Cox EN). While flexible models introduced greater variability, they were more effective in capturing nonlinear exposure-response relationships. These findings underscore the value of model flexibility in complex environmental mixture analyses, despite the efficiency trade-offs involved. We also found that optimal model selection may depend on the choice of estimand as well as whether the primary aim is to estimate the overall mixture effect or effects of individual exposures.

Our approach has many strengths compared to prior mixture analyses for survival outcomes. For example, prior analyses have used alternative study designs or metrics to quantify survival outcomes as binary or continuous. We utilize a time-to-event model to more appropriately account for the nature of incident of event [31–33]. We also preserved the dimensionality and continuous structure of the exposures rather than dichotomizing individual exposure concentrations or applying unsupervised learning methods to identify exposure subgroups, allowing us to more accurately capture the dynamic exposure-outcome relationships [34, 35].

While our study offers valuable insights, several limitations merit consideration. Firstly, although we attempt to characterize the selected methods based on an array of characteristics, there are other modeling features relevant to mixtures analyses that are not considered in detail. For example, variable selection and shrinkage methods mitigate the adverse impacts of multicollinearity commonly observed across environmental mixtures. Of the methods we consider, only BART, Cox EN and MARS perform variable selection, yet our assessment does not include the evaluation of the accuracy with which each method conducts variable selection. Furthermore, while variable selection is a desirable feature in environmental mixture studies for estimation, it presents challenges for measuring uncertainty. While the non-parametric bootstrap is commonly used in practice, it underestimates uncertainty due to its failure to account for the inherent uncertainty in the variable selection process conducted using observed data. For example, bootstrap sampling for the Lasso estimator has been shown to introduce bias that remains asymptotically when true coefficient values are close to or exactly zero [36]. The Cox EN model faces similar issues with its bootstrap confidence intervals. To mitigate these challenges and provide more reliable confidence intervals, multiple post-selection inference approaches have been proposed [37]. Alternatively, Bayesian methods straightforwardly calculate uncertainty via the estimated posterior distribution of the estimand, circumventing post-selection inference challenges. One such method, Bayesian Kernel Machine Regression (BKMR), has been previously adapted to discrete-time survival analysis in a smaller datasets (*n* = 1,171) [6]. However, in our attempts to apply BKMR, we were unable to achieve convergence after extended runtimes on a high-performance computing cluster, highlighting its current computational limitations for larger cohorts. While a Fast BKMR version has recently been developed, it is currently only available for continuous outcomes [38]. Our inability to implement otherwise widely used mixture methods to the survival setting reinforces the contribution of our work. Improving the computational feasibility of flexible modeling approaches, particularly Bayesian methods, for environmental mixtures with survival outcomes remains an important direction for future research.

Several other survival-specific aspects warrant future exploration, such as the choice of time intervals for the discrete-time survival analysis approach. We used five time bins to balance information retention with computational feasibility, as increasing the number of intervals increases the size of the augmented dataset, leading to longer runtimes. Calendar-based intervals, rather than quantiles of observed times, could also be considered. We chose the latter because we relied on the built-in data augmentation function in the BART R package. This approach also helps to ensure sufficient information in each interval, improving power and stability, though potentially at the cost of interpretability. Our comparison of methods’ performances when *R* = 5 compared to when *R* = 10 found that this choice had little impact, though this may not be the case in other settings, particularly when the proportional hazards assumption is violated. Other survival-specific considerations include evaluating how censoring affects model bias and assessing each method’s ability to accommodate time-varying effects.

Previous evaluations of environmental mixture methods have concluded that no single statistical method consistently outperforms others and the optimal choice depends on the scientific context or pre-analysis hypotheses [9–11, 39, 40]. Others have highlighted the advantages of flexible methods [8]. We extend this growing area of to the survival outcome setting, demonstrating how to apply flexible methods to capture complex mixture relationships on a survival outcome and confirming their advantages despite higher computational demands. Factors such as sample size, follow-up duration, exposure correlations, and the underlying functional forms can influence model performance. Given this variability, we recommend assessing robustness of results by applying multiple statistical methods. This work underscores the importance of developing new or extending existing methods to more effectively estimate the effects of environmental mixtures in the context of survival outcomes.

## Supplementary Information

Below is the link to the electronic supplementary material.

ESM 1 (DOCX 993 KB)

## Data Availability

The data were collected, analyzed, and reported under agreements made with the sovereign tribal nations that have partnered in this research, which precludes commonly accepted modes of data sharing. Requests to access the dataset from qualified researchers trained in human subject confidentiality protocols may be sent to the Strong Heart Study CoordinatingCenter at https://strongheartstudy.org/. Requests will be reviewed by tribal research partners before data may be released. This policy is consistent with the NIH Policy for Data Management and Sharing: Responsible Management and Sharing of American Indian/Alaska Native Participant Data.
