# Supplementary material for: Comparison of Methods for Analyzing Environmental Mixtures Effects on Survival Outcomes
Source: Curr Environ Health Rep. 2025 Nov 1;12(1):40. doi: 10.1007/s40572-025-00500-y (PMC12578748; doi:10.1007/s40572-025-00500-y)
Supplement: Supplementary file 1 — (DOCX 993 KB) [file 40572_2025_500_MOESM1_ESM.docx]

**Supplemental Material**

**Comparison of methods for analyzing environmental mixtures effects on survival outcomes**

Melanie Mayer^1*^, Arce Domingo-Relloso^2^, Marianthi-Anna Kioumourtzoglou^3^, Ana
Navas-Acien^3^, Brent A. Coull^4^, Linda Valeri^2^

^1^Department of Biostatistics, Epidemiology, and Informatics, Perelman School of Medicine, University of Pennsylvania, Philadelphia, PA, USA

^2^Department of Biostatistics, Columbia Mailman School of Public Health, New York, New York, USA

^3^Department of Environmental Health Sciences, Columbia Mailman School of Public Health, New York, New York, USA

^4^Department of Biostatistics, Harvard T.H. Chan School of Public Health, Boston, Massachusetts, USA

^*^Corresponding author: Melanie Mayer, [Melanie.Mayer@PennMedicine.upenn.edu](mailto:Melanie.Mayer@PennMedicine.upenn.edu), 423 Guardian Drive, Philadelphia, PA 19104

Abbreviations and Acronyms:

BART – Bayesian Additive Regression Trees

BMI – Body mass index

Cox PH-ps – Cox Proportional Hazards with penalized splines

CV – Cross-validation

EN – Elastic net

GPR – Gaussian Process Regression

HR – Hazard ratio

IQR – Interquartile range

MARS – Multivariate Additive Regression Splines

MISE – Mean integrated squared error

ML – Machine learning

PH – Proportional hazards

RMSE – Root mean squared error

SD – Standard deviation

SPD – Survival probability difference

**Supplemental Material S1**

*Additional Quantities of Interest*

Although the primary focus of this study was to analyze the effect of an environmental mixture on survival time outcomes, we also chose to include estimates of the effect of an individual mixture component and the interaction among two mixture components on survival outcomes to acknowledge the diverse goals of environmental mixture studies. Consider the hazard function, $\lambda(t)= \lim_{\Delta t\to0} Pr\left( t\leq T\leq t+\Delta t | T>t \right)/\Delta t$, and the survival probability, $S\left( t \right)=P(T>t)$, both of which are a function of time, $t$. To estimate the effect of a subset of the exposure set $\{M_{1}, ..., M_{J}\}$ on a survival outcome, we use $\lambda_{\boldsymbol{j}\subset\left\{ 1,\ldots,J \right\}}^{q_{1},\ldots, q_{\left| j \right|}}(t|\boldsymbol{M}_{-\boldsymbol{j}}, \boldsymbol{C})$ and $S_{\boldsymbol{j}\subset\left\{ 1,\ldots,J \right\}}^{q_{1},\ldots, q_{\left| j \right|}}(t|\boldsymbol{M}_{-\boldsymbol{j}}, \boldsymbol{C})$ to denote the hazard and survival probability, respectively, at time $t$*,* when exposed to environmental mixture components who’s indices are in $\boldsymbol{j}$ at their respective $q_{1}^{th}, \ldots, q_{|\boldsymbol{j}|}^{th}$ percentiles, conditional on all other metals $\boldsymbol{M}_{-\boldsymbol{j}}$ and all confounders $\boldsymbol{C}$. We quantify the effect of an exposure to an individual metal, $M_{j}$, on the survival outcome as the HR and the SPD for an IQR change in exposure to metal $M_{j}$, holding all other metals and confounders constant (e.g. at their median). These quantities are formulaically expressed in Table S1.

To better understand whether an interaction exists between metals, the interaction is quantified on the multiplicative scale. Define

$$HR_{j, j^{'}}^{q, q^{'}}= \frac{\lambda_{j,j^{'}}^{q,q^{'}}(t|\boldsymbol{M}_{\boldsymbol{-\{j,}\boldsymbol{j}^{\boldsymbol{'}}\boldsymbol{\}}},\boldsymbol{C})}{\lambda_{j,j^{'}}^{q,q}(t|\boldsymbol{M}_{\boldsymbol{-\{j,}\boldsymbol{j}^{\boldsymbol{'}}\boldsymbol{\}}},\boldsymbol{C})}$$

where the subscript specifies the two metals we are interested in detecting an interaction between, $M_{j}$ and $M_{j^{'}}$ where $j\neq j^{'}$. The superscript specifies the percentiles we are interested in detecting an interaction at, this can be thought of as high versus low concentrations of these exposures. The multiplicative interaction can then be defined as ${HR_{j,j^{'}}^{q^{'},q^{'}}}/{HR_{j,j^{'}}^{q^{'},q}HR_{j,j^{'}}^{q,q^{'}}}$, or the excess hazard in the presence of high levels of metals $M_{j}$ and $M_{j^{'}}$ jointly compared to high levels of each metal individually.^1^ We estimated these by setting $q=25$ and $q'=75$ and plugging in the estimated hazards into the equation above, while setting the confounders and other metals to their median values. The mathematical formulation of this quantity can be seen in Table S1.

Interaction effects can also be quantified on the additive scale for survival outcome models via the relative excess risk due to interaction ($RERI=HR_{j, j^{'}}^{q^{'},q^{'}}-HR_{j,j^{'}}^{q^{'}, q}-HR_{j,j^{'}}^{q,q^{'}}+1$).^2^ However, they wouldn't be properly defined for models that do not allow for interactions, such as the Cox PH Model without interactions, because while the product term is modeled such that it is zero between the two metals of interest, the estimated RERI may still differ from zero. On the other hand, the multiplicative interaction will be one when no interaction is included in the model. We therefore chose not to include the RERI.

**Supplemental Material S2**

*Mathematical Forms and Computational Details of Statistical Methods*

Here we include additional details on the computational procedure conducted by some of the selected methods. See Table S2 for a summary of the hyper-parameters required for each method and the average run time for each modeling method.

To estimate the quantities of interest using the discrete time approach for survival outcomes, one can estimate the survival probability at discretized time bin $r$, for $r=1,\ldots,R$, as $S(t_{\left( r \right)} |\boldsymbol{m},\boldsymbol{c}) = Pr(T > t_{\left( r \right)} | \boldsymbol{m},\boldsymbol{c}) =\Pi_{l=1}^{r}(1 - pr(t_{\left( l \right)}, \boldsymbol{m},\boldsymbol{c}))$ and the hazard as $\lambda(t_{\left( r \right)} | \boldsymbol{m},\boldsymbol{c})=pr(t_{\left( r \right)}, \boldsymbol{m},\boldsymbol{c})/(t_{\left( r \right)}-t_{(r-1)})$.^3^ Here, $pr(t_{\left( r \right)}\boldsymbol{,m},\boldsymbol{c)}$ denotes the probability of experiencing the event within time bin $t_{(r)}$, with fixed exposures at $\boldsymbol{m}$ and confounders $\boldsymbol{c}$ conditional on no previous event.

To estimate the confidence intervals for all non-Bayesian models, we used the nonparametric bootstrap. While some models could rely on the asymptotic distributions of the effect estimates to estimate confidence intervals, we chose to use the nonparametric bootstrap instead to be more consistent across methods. The bootstrap confidence interval may be more accurate in the presence of model misspecification or a small sample size, both of which are common in environmental mixtures studies. We randomly sampled 500 datasets with replacement of size $n$ (where $n$ is the sample size) from the original dataset and estimated each estimand of interest for each bootstrap sample. The 2.5^th^ and 97.5^th^ percentiles of these 500 estimated quantities were used as the lower and upper limits, respectively, of the confidence intervals.

*Traditional Cox Proportional Hazards Model (Cox PH).* The hazard function is modeled as

$$\lambda\left( t;\boldsymbol{m},\boldsymbol{c} \right)=\lambda_{0}\left( t \right)exp\{\Sigma_{j=1}^{J}\beta_{j}m_{j}+\Sigma_{l=1}^{L}\gamma_{l}c_{l}\},$$

for $J$ metals and $L$ confounders.^4^ The coefficients are estimated via the partial likelihood method.

*Cox Proportional Hazards Model with Penalized Splines (Cox PH-ps)*. The hazard function has the form

$$\lambda\left( t;\boldsymbol{m},\boldsymbol{c} \right)=\lambda_{0}(t)exp\{\Sigma_{j=1}^{J}f_{j}(m_{j}) +\Sigma_{j\neq j^{'}}f_{j,j^{'}}(m_{j}, m_{j^{'}}) +\Sigma_{l=1}^{L}\gamma_{l}c_{l}\},$$

where $f_{j}$ and $f_{j,j^{'}}$ are smooth functions estimated via penalized splines through tensor product smoothers. While we modeled all individual metals (e.g. $f_{1}(m_{1})$) and two-way interactions between metals (e.g. $f_{1,2}(m_{1}, m_{2})$) smoothly, the covariates were modeled linearly (e.g. $\gamma_{1}c_{1}$).

*Cox Elastic Net (Cox EN).* EN constrains the estimated coefficients by maximizing the likelihood subject to the constraint $\omega\Sigma\left| \beta_{j} \right|+ \left( 1-\omega\right)\Sigma\beta_{j}^{2}\leq d$.^5^ $\omega\in[0,1]$ is the mixing parameter, which specifies the ratio of the constraint pertaining to the L1 ($\Sigma|\beta_{j}|$) and L2 ($\Sigma\beta_{j}^{2}$) penalties. To estimate $\boldsymbol{\beta}$, the optimization problem subject to the given constraint can be written in its Lagrangian formulation and we consider the regularization parameter $\kappa\in[0,\infty)$. If $\kappa=0$ then this is equivalent to the traditional Cox PH model. The larger $\kappa$, the stronger the regularization and the more the coefficients will be shrunk towards zero. In our model, the coefficients pertaining to the confounders ($\boldsymbol{\gamma}$) were not included in the constraint.

CV was used to find the optimal choices for $\omega$ and $\kappa$. The R package *glmnet* (version 4.1-3) incorporates k-fold CV when estimating the Cox EN coefficients. The cv.glmnet command returns a value of $\kappa$ for a fixed $\omega$. A pathwise solution via cyclic coordinate descent is used, where $\kappa$ ranges from sufficiently large enough for $\boldsymbol{\beta}=0$, and decreases until near the unregularized solution ($\kappa=0$). To find the optimal $\omega$, a grid of potential values was used (see Table S2). At each value of $\omega$, the optimal $\kappa$ is determined through the CV described. The final model is the combination of $\omega$ and $\kappa$ which maximizes $l(\boldsymbol{\beta}_{-i} (\omega,\kappa))- l_{-i}(\boldsymbol{\beta}_{-i}\left( \omega,\kappa\right))$, where $l_{-i}$ is the log partial likelihood excluding part $i$ of the data and $\boldsymbol{\beta}_{-i}(\omega,\kappa)$ is the optimal $\boldsymbol{\beta}$ for the non-left out data. CV was performed for each bootstrap sample when estimating the corresponding confidence interval.

*Multivariate Adaptive Regression Splines (MARS).* The MARS algorithm creates a piecewise linear model.^6^ $h_{p}(\boldsymbol{m},t)$ is a piecewise linear function of an exposure (or interaction of piecewise linear functions) included in the final model. For example, $h_{p}(\boldsymbol{m},t)=\left( m_{j}-\circ\right)_{+}$ is a piecewise linear function of metal $m_{j}$ starting from the knot value $\circ$. The “+” indicates that we are referring to the positive section, such that $h_{p}\left( \boldsymbol{m},t \right)=m_{j}-\circ$ when $m_{j}>\circ$ and 0 otherwise. To incorporate interactions, $h_{p}(\boldsymbol{m},t)$ can also be a product of piecewise linear functions, such as $h_{p}\left( \boldsymbol{m},t \right)=\left( m_{j}-\circ_{1} \right)_{+}\times\left( m_{j^{'}}- \circ_{2} \right)_{+}$. CV is performed to optimize the maximal degree of interactions ($D$) and the number of terms retained ($P$) in the final model, which has the form

$$f\left( \boldsymbol{m,c},t \right)=\Sigma_{p=1}^{P}\beta_{p}h_{p}(\boldsymbol{m},t) +\Sigma_{l=1}^{L}\gamma_{l}c_{l}$$

We included the confounders in the model linearly, as seen in $\Sigma_{l=1}^{L}\gamma_{l}c_{l}$ and used the logit link function for our MARS model adaptation to discrete time survival analysis. We used the R package *earth* (version 5.3.1) and perform 5-fold CV for $D$ (using grid of values: [1,2]) and $P$ (using grid of values: [2, 4, …, 30]) using the R package *caret* (version 6.0-90). The area under the receiver operating characteristic (ROC) curve is used for goodness of fit in the CV procedure. CV was performed for each bootstrap sample when estimating the corresponding confidence interval.

*Bayesian Additive Regression Tree (BART).* BART is a nonparametric Bayesian sum of trees approach. Let $h_{p}(\boldsymbol{m}_{i}\boldsymbol{,}\boldsymbol{c}_{i}, t;U_{p},V_{p})$ represents an individual tree where $p=1,\ldots, P$. $U_{p}$ denotes the $p^{th}$ tree’s terminal nodes and $V_{p}$ denotes its predicted outcome value from the terminal nodes. The function form for the sum of trees approach is

$f(\boldsymbol{m}_{i},\boldsymbol{c}_{i},t) =\Sigma_{p=1}^{P}h_{p}(\boldsymbol{m}_{i},\boldsymbol{c}_{i},t;U_{p},V_{p})$.

To estimate these trees, a Bayesian approach is used where the prior follows $pr((U_{1},V_{1}),\ldots,(U_{P},V_{P}))=\Pi_{p} pr(U_{p})[\Pi_{i} pr(\mu_{ip} |U_{p})]$. $pr(U_{p})$ is specified by (i) the probability that a depth $d$ node is nonterminal, (ii) the distribution on the splitting variable assignments at each interior node, and (iii) the distribution on the splitting rule assignment in each interior node, conditional on the splitting variable. (i) is given by $a\left( 1+d \right)^{-b}$, $a\in(0,1)$ and $b\in[0,\infty)$. The software default values for the hyperparameters are $a=0.95$ and $b=2$. Using these values, trees with $1, 2, 3, 4$ and $\geq5$ terminal nodes receive prior probability of $0.05, 0.55, 0.28, 0.09$ and $0.03$, respectively, such that individual trees are kept small. A uniform prior is placed on the choice of splitting variable at each node, and a discrete uniform prior is specified for the splitting values. For $p(\mu_{ip}|U_{p})$ we consider $\mu_{ip}\sim N(0,\sigma_{\mu}^{2})$ where $\sigma_{\mu}= 3.0/(k\surd P)$. These priors shrink $\mu_{ip}$ toward zero, limiting the effect of individual tree components. As $k$ and/or the number of trees $P$ increases, this prior will become tighter and apply greater shrinkage to $\mu_{ip}$.

The default parameters provided by the R package *BART* (version 2.9.0) are $a=0.95$, $b=2$, $k=2$ and $P=50$, these have been shown to perform well. Thus, we choose to use them in all our analyses to aid run time at the expense of potential improvements to model performance. Alternatively, the value of $k$ and $P$ may be chosen by CV. To sample from the posterior distribution, a Bayesian back-fitting Markov chain Monte Carlo algorithm was used. 1000 draws from the posterior were generated after a burn-in of 250 draws. Due to the repetition between observations of the augmented dataset, we are more likely to run into issues with high auto-correlation, thus we used thinning of 5 between each returned value.

**Supplemental Material S3**

*Simulation Study Setup*

Here we describe how the data for the simulation study were produced. For all scenarios, three confounders were simulated: $sex\sim Bernoulli(p=0.59)$, $BMI\sim N(3.39,0.19$), and $age\sim N(56.13, 8.10)$. Let $\boldsymbol{C}=\{Sex,BMI,Age\}$ denote the set of simulated confounders.

The metals were simulated as a linear function of these confounders, previously simulated metals, and noise. For example:

$$M_{1}=\Phi_{0,1}+\Phi_{sex,1}sex +\Phi_{BMI, 1}BMI +\Phi_{age,1}age+N(0,\sigma_{1}^{2})$$

$$M_{2}=\Phi_{0,2}+\Phi_{sex,2}sex+\Phi_{BMI,2} BMI+\Phi_{age,2} age+\Phi_{1,2}M_{1}+N(0,\sigma_{2}^{2})$$

$$...$$

$$M_{J}=\Phi_{0,J}+\Phi_{sex,J} sex+\Phi_{BMI,J}BMI+\Phi_{age,J}age+\Phi_{1,J}M_{1}+\Phi_{2,J}M_{2}+ ...+\Phi_{J-1,J} M_{J-1}+N(0,\sigma_{J}^{2})$$

$J$ is the number of metals simulated for the metal mixture set $\boldsymbol{M}=\{M_{1},M_{2},... , M_{J}\}$. This approach leads to moderate correlations ranging from around 0.01 to 0.27. Panel B of Figure S1 shows the correlations across the metal exposures from a simulated dataset used in our simulation study for the base-case scenario.

It is also common in environmental mixture studies to have higher correlations across exposures. For example, many exposures may come from the same source. Subjects with higher exposure to the source will thus have higher concentrations of multiple exposures. Therefore, there may also be interest in assessing methods’ performances in the presence of higher correlations. To consider the scenario where there are higher correlations across metals, the exposures were simulated using larger coefficients ($\Phi_{j,j^{*}}$for $j\neq j^{*}$), each original coefficient was increased by one. An example of the resulting correlations across the simulated metal exposures for a simulated dataset replicating the higher correlation setting can be seen in Panel C of Figure S1, where the correlations range from 0.42 to 0.64.

The time-to-event outcome was simulated as $t\sim Weibull(\alpha(\boldsymbol{m,c}),f(\boldsymbol{m,c}))$, where

$$S(t;\boldsymbol{m,c})= exp\{-({\frac{t}{f\left( \boldsymbol{m,c} \right)})}^{\alpha\left( \boldsymbol{m,c} \right)}\}$$

$$\lambda(t;\boldsymbol{m,c}) = \frac{\alpha\left( \boldsymbol{m,c} \right)}{f\left( \boldsymbol{m,c} \right)}\left( \frac{t}{f\left( \boldsymbol{m,c} \right)} \right)^{(\alpha\left( \boldsymbol{m,c} \right)-1)}$$

Two censoring random variables were used, $C_{1}\sim Uniform(0,100)$ and $C_{2}\sim Uniform\left( 16,20 \right)$, such that $C\sim min(C_{1},C_{2})$. This setting replicates the setting where there is continuous uniform censoring followed by increased uniform censoring towards the end of follow-up time. All scenarios approximate 67% of censoring, where observations do not experience the event by the end of their follow-up time.

We controlled whether the PH assumption holds through $\alpha\left( \boldsymbol{m,c} \right)$ and the functional form of the effect of $\boldsymbol{C}$ and $\boldsymbol{M}$ on the outcome through $f\left( \boldsymbol{m,c} \right)$. For all scenarios, the confounders $\boldsymbol{C}$ were considered to have a log-linear effect on the outcome while the mixture components are log-non-linear, such that $f(\boldsymbol{m},\boldsymbol{c}) = exp\{\Sigma_{k=1}^{3}\gamma_{k}c_{k} -1.55\left( m_{1}+2 \right)^{\frac{1}{4}}+\frac{8}{1+e^{3.3m_{3}-7}}+1.5(m_{4}+{3.5}^{2}(m_{5}+1)\}$.

Note that only four components of $\boldsymbol{M}$ were included in $f\left( \boldsymbol{m,c} \right)$, such that $m_{2}$ is not associated with the outcome. We simulate data where the proportional hazards assumption holds by setting $\alpha\left( \boldsymbol{m,c} \right)=1$. For the non-proportional hazards scenario, the outcome was simulated using $\alpha\left( \boldsymbol{m,c} \right)=0.7+0.1m_{1}+0.1m_{3}+0.1m_{4}+0.1m_{5}$. For the scenario with a 10-dimensional mixture, the same functional form is used for $f(\boldsymbol{m},\boldsymbol{c})$, such that 6 of the 10 mixture components have no effect on the outcome.

**Supplemental Material S4**

*Additional Simulation Results*

*Power with and without censoring.* Censoring is an important and complex topic which arises when considering a survival outcome. For all simulations we generated, we roughly set ~70% of observations as censored. Having such a high percentage of observations censored, however, likely greatly affected our results. This causes reduction in the effective sample size and can lead to a reduction in power. The more flexible methods particularly tend to suffer from lack of power and require a larger sample size to provide more stable estimates. We compare the base-case scenario with and without censoring.

In Figure S2 we compare each method's ability to detect a significant effect (or power) for the individual metal and the metal mixture on both the HR and SPD scale. The true individual metal effect (HR = 1.14, SPD = -0.016) was smaller compared to the complete mixture effect (HR = 2.40, SPD = -0.089), thus we’d expect the models to have higher power for estimating the mixture compared to the individual exposure. This is in fact what we see, with all methods achieving perfect power (1.0) when estimating the mixture effect except for the ML style methods, MARS and BART (Figure S2, panels in the 2^nd^ column). This is expected, given that flexible methods typically have higher variability, wider confidence bands, and thus lower power. The power does increase for both methods in the absence of censoring however, with MARS even achieving perfect power in the absence of censoring.

For estimating the individual exposure effect (Figure S2, panels in the 1^st^ column), however, all methods other than MARS have lower power, with large increases in the absence of censoring. BART has very low power, even in the absence of censoring. Given how flexible BART is, we may need a much larger sample size to significantly improve its power and be able to detect a small but significant effect. Unexpectedly, MARS achieves the highest power for the individual effect, and even has perfect power in the absence of censoring. For the proportional hazards methods, removing censoring makes a drastic difference, especially for the models where interaction terms are included. Given that power does tend to increase when there is a larger true effect size or lower censoring, sufficient power may only be viable for larger effects, large sample size, or low censoring. One might want to consider what a realistic effect size might be or their effective sample size when choosing a model.

*Results at varying follow-up times.* Figure S3 shows how the models vary in performance when estimating the mixture effect at varying times for the base-case scenario. Since the proportional hazard assumption holds for this scenario, the HR is the same at both time points and the estimated HR for the models which make the proportional hazards assumption is the same at both time points. Thus, as expected, their performance when estimating the HR is independent of the time point of interest. However, we see an increase in bias and lower coverage in the estimates from these models, other than Cox PH-ps, when estimating the SPD at the earlier time point compared to the later time point. At 10 years it’s likely that most observations had not experienced the event yet, leading to greater bias at this earlier time-point. However, the variances are lower at the earlier time, resulting in low coverage. Alternatively, Cox PH-ps retains good performance at this earlier time point relative to the later timepoint.

For the discrete-time survival models, MARS and BART, coverage remains above 95% for 10-year estimates for both estimands. For the most part, we also see a reduction in bias at this earlier time point, other than for the MARS SPD estimate, and a reduction of standard deviation. Methods’ performances relative to one another seems to be mostly unaffected by the choice in time to estimate the effect at.

*Results using K=10 time bins.* Figure S4 shows how the performance of the discrete-time survival analysis methods for estimating the HR and SPD for an interquartile range change in each component of a mixture for the base-case scenario changed when using different number of bins (K) when running the model. BART and MARS appear to perform similarly when using 5 versus 10 time bins. Specifically, the coverage probability does not change, and the standard deviations don’t vary much. For the SPD, bias also appears to be stable. However, we actually see a slight increase un bias with an increase in K for the estimates from the MARS model, from 0.06 from the K=5 model to 0.11 from the K=10 model. We may not see a big improvement in performance when we increase K due to the nature of the discrete time survival analysis, where we can estimate the HR and SPD at any time point if we assume constant hazard, which holds in this scenario. Thus, we may just be adding more noise by using a higher number of bins and running the model on a larger augmented data set without providing much more information. The average run time for the BART model did not increase with the increase in number of bins, taking an average of 43.7 minutes when K=5 and 44.5 minutes when K=10. MARS, on the other hand, increased from an average of 3.96 hours when K=5 to 6.48 hours when K=10. The choice in K should be made with consideration to the plausibility of the constant hazards assumption and your computational feasibility.

*Exposure-response curves for various non-linear functions.* Figure S5 plots the mean integrated squared error (MISE) for the exposure-response curves for a single metal under various simulation scenarios. In relation to Figure 2 from the main text, these pertain to the survival curve for Metal 3. As expected, the more flexible methods (Cox PH-ps, MARS, and BART) achieve a lower MISE across all scenarios compared to the more constrained models. The latter achieve only a slightly lower MISE for the models where interactions are included in the model, and all see large increases in MISE in the higher correlation setting. The more flexible methods are more resilient across scenarios, particularly BART, which achieves a consistent MISE across all scenarios. This emphasizes the need to choose a flexible model when the goal of the researcher is to estimate the exposure-response curve and there is reason to believe non-linearities exist.

**Supplemental Material S5**

*Real-world data application: Strong Heart Study*

We further demonstrate our proposed framework in a real-world setting. The Strong Heart Study (SHS) is a cohort study that aims to assess the risk factors associated with incidence of cardiovascular disease (CVD) in American Indians.^7^ Previous SHS studies have examined associations between individual metal exposures and incident CVD, evaluating how other metals influence the primary exposure of interest rather than considering their joint effects. Potential nonlinear and interactive effects have been found, underscoring the value of flexible modeling approaches. Urinary As and Cd levels have been found to be prospectively associated with incident clinical CVD.^8,9^ The exposure-response function for the association of Se with incident CVD was found to be U-shaped, with both lower and higher levels of urinary Se associated with excess CVD risk.^10^ Additionally, an interaction between W and Mo has been reported, where tungsten was associated with higher CVD risk only at low molybdenum levels.^11^ Despite earlier work identifying mixture patterns, no studies to date have quantified the joint effect of the mixture on incident CVD in this population.^12^ To account for the potential interplay of these exposures on CVD in the SHS, we applied all seven modeling approaches to estimate the joint effect of a six metals/metalloids mixture (arsenic, cadmium, molybdenum, selenium, tungsten, and zinc) on incident CVD in the SHS, illustrating how the proposed framework can be implemented in practice.

The SHS population has been described in detail previously.^7^ Briefly, between 1989 and 1991, 4,549 men and women aged 45–75 from 13 tribes located in Arizona, Oklahoma, North Dakota, and South Dakota were recruited to participate in a prospective cohort study to investigate CVD and its risk factors in American Indian adults. Participants were followed for clinical events through 2017. One community withdrew their consent to participate in further research in 2016, leaving 3,516 participants. Additionally, we excluded 251 individuals with CVD at baseline, 434 without urinary metal measurements, and 103 who were missing other variables of interest, leaving an analytical sample of 2,728 individuals. The study protocol was approved by the institutional review boards of the Indian Health Service, the participating institutions, and the participating tribes. Each participant provided individual written informed consent.

Metals were measured in urine at the baseline visit. Detailed analytical methods can be found in Scheer et al.^13^ For arsenic, speciation was conducted using high performance liquid chromatography coupled with inductively coupled plasma mass spectrometry. The sum of inorganic and methylated arsenic species was used as the biomarker of toxic arsenic exposure. The limits of detection (LOD) for As, Cd, Mo, Se, W, and Zn were 0.1, 0.015, 0.1, 2.0, 0.005, and 10.0 $\mu$g/L and the percentage of samples below the LOD in the analytical sample was 0%, 0.037%, 0%, 0%, 1.2%, and 0%, respectively. Urinary metal/metalloid concentrations, jointly referred to as metals throughout for simplicity, below the LOD were replaced with the LOD divided by the square root of two. To account for urine dilution, urinary metal concentrations were divided by urine creatinine levels and expressed as $\mu$g/g creatinine. Urinary levels of the metals were skewed, thus were log-transformed for all statistical analysis.

CVD endpoints were assessed by questionnaire, tribal records, Indian Health Service hospital records, death certificates, and direct contact. All deaths and CVD outcomes were reviewed by the Morbidity and Mortality Review Committee based on the World Health Organization criteria. Follow-up through 2017 was 99.8% complete for mortality and 99.2% complete for nonfatal events. Incident CVD was defined as definite or possible fatal or non-fatal congenital heart disease (CHD), stroke, or heart failure.^7,14^ Survival time was defined as the time from start of follow-up (1989-1991) and was calculated as the difference between age at the date of the baseline examination and age at the date of the cardiovascular event, age at the date of death, or age at end of follow-up, whichever occurred first.

Trained and certified nurses and medical examiners collected information on baseline covariates. To adjust for confounding, the following confounders were included in all models: baseline age, sex, education level (none, some, or high school), smoking status (never, former, or current), body mass index (BMI) (kg/m^2^), kidney function, and arsenobetaine. Kidney function was quantified as the estimated glomerular filtration rate (eGFR) calculated from creatinine, age, and sex using the Chronic Kidney Disease Epidemiology Collaboration formula.^15^ We included eGFR due to its known impact with CVD and relationship with metal excretion in urine. Arsenobetaine was included as a marker of seafood arsenicals. Confounders were entered linearly into all models where the confounders/outcome relationship could be specified.

Descriptive statistics for the baseline confounders and metal mixture components, including their IQRs, are presented in Table S3. Urinary concentrations of the metals were weakly to moderately correlated, with Spearman correlation coefficients ranging from 0.01 to 0.26. The correlation matrix can be found in Figure S1, Panel A. A total of 904 participants (33.14%) developed incident CVD by the end of follow-up time. Although all models included the full set of metals/metalloids and two-way interactions (where applicable), we focus on a select subset of results. In addition to examining the overall mixture effect, we highlight the estimated association of Se with CVD, motivated by previous findings underscoring the need for flexible modeling approaches for more accurate estimation, as well as its interaction with W, given emerging evidence that W may enhance the toxicity of other exposures.^10,16^ We estimated the quantities of interest at 10 years and the 80^th^ percentile of the observed follow-up time, which was 18.4 years. For the discrete survival-time models, time was discretized into $R=5$ bins.

Diagnostic plots for convergence assessment of the BART model can be seen in Figure S6. The trace plots of the posterior predicted probabilities for a random subset of training observations show stable sampling behavior with no visible drift or trend, indicating convergence of these BART chains. As expected, given the rarity of the CVD outcome, the posterior probabilities were generally concentrated in the lower range (e.g., <0.4). Using the conventional -2 to 2 range (denoted by dashed red lines) as a 95% confidence threshold, the Geweke diagnostic plot with Z-scores based on a test for equal means between the first 10% and last 50% of each Markov chain show that most values fall within these bounds, suggesting convergence. While some observations do exceed these bounds, the quantity is reasonable given the large number of observations. Lastly, the plot displaying the autocorrelation function (ACF) plot of posterior samples for a random subset of 100 subjects shows the autocorrelations dropping rapidly after the first few lags, indicating low serial dependence and appropriate thinning. Taken together, these diagnostics suggest satisfactory convergence of the BART model’s posterior distribution.

We estimated the HR and SPD for an IQR change in Se and the overall metal mixture across the different modeling methods, shown in Figure S7. For methods assuming proportional hazards, the HR is constant over time, and thus these estimates remain unchanged at both time points. All methods found a significant, harmful effect (HR>1, SPD <0) of increased Se and metal mixture concentrations on incident CVD at both time points. For Se, the estimated HR at the 80^th^ percentile of time ranged from 1.28 (1.17, 1.41) to 1.59 (1.17, 2.83); for the metal mixture, from 2.01 (1.77, 2.49) to 3.15 (1.47, 7.92). For both, Cox EN with interactions produced the smallest estimates, and BART the largest. The estimated SPD at the 80^th^ percentile of time ranged from -0.06 (-0.09, -0.04) to -0.09 (-0.18, -0.03) for Se, and -0.18 (-0.24, -0.15) to -0.24 (-0.40, -0.1) for the mixture, where again the smallest estimates were estimated via Cox EN with interactions and the largest via BART. Similar trends were observed at 10 years, with all models finding significant effects, though often slightly smaller compared to the later time point. Our findings are consistent with the simulation study results, where more flexible methods tended to produce wider confidence bands. While we cannot assess bias in a single real-world dataset, the simulation results suggest that these wider confidence intervals result in better coverage and thus more likely capture the true value.

Figure S8 shows the methods’ point estimates and their confidence intervals for the multiplicative interaction between Se and W on incident CVD. Cox PH and Cox EN assume no interaction, thus, will always calculate a null effect of 1. However, all the other models also find null effects, indicating that the effect of both Se and W at their ${75}^{th}$ percentile together is no different than the product of the effects of Se and W considered separately at their ${25}^{th}$ and ${75}^{th}$ percentile.

In summary, we analyzed the association between a six-component mixture and incident CVD. We included both toxic (As, Cd, W) and essential (Mo, Se, Zn) elements, many of which have been previously linked to CVD outcomes through complex biological pathways. For instance, As and Cd are heavy metals that have been found to be associated with CVD even at low concentrations, potentially via methylation dysregulation that disrupts cellular processes.^17–24^ Elevated W biomarkers have also been tied to increased CVD risk, potentially by replacing Mo, an essential metal, at protein binding sites and increasing oxidative stress.^11,25^ These complex, interactive, and sometimes non-monotonic relationships, such as the previously reported U-shaped association between Se and CVD risk in the SHS, underscore the importance of using models that can flexibly capture effects from multiple, continuous exposures.^10^ Across all methods, we found a significant positive association, suggesting that higher concentrations of urinary metals/metalloids are associated with increased CVD risk in this population. The consistency in the direction of estimated effects across methods reassures us of the robustness of our findings; however, variability in their magnitudes emphasizes how model choice can influence clinical interpretability and potentially inform prevention strategies. One should exercise caution in interpreting findings based on a single modeling approach and ideally assess whether results are robust across different modeling approaches.

Interpreting our findings requires consideration of potential limitations, such as complexities associated with the measurement of metal exposure via urine. Previous studies have found that, although Mo, Se, and Zn are essential metals that serve important biological functions, urinary biomarkers of these elements have been unintuitively positively associated with CVD. This could be due to urinary traces reflecting metabolic dysregulation rather than excess intake.^26–28^ For example, while Zn is essential for insulin regulation, excess urinary Zn is often produced by individuals with diabetes, a major CVD risk factor.^29–31^ However, our finding could have significant public health implications, underscoring the potential need to reduce metal exposures in American Indian communities, where exposure levels are generally higher compared to the broader U.S. population, and merits further investigation.

**References**

1. VanderWeele TJ, Knol MJ. A Tutorial on Interaction. *Epidemiol Methods*. 2014;3(1):33-72. doi:10.1515/em-2013-0005

2. VanderWeele TJ. Causal interactions in the proportional hazards model. *Epidemiol Camb Mass*. 2011;22(5):713-717. doi:10.1097/EDE.0b013e31821db503

3. Sparapani RA, Logan BR, McCulloch RE, Laud PW. Nonparametric survival analysis using Bayesian Additive Regression Trees (BART). *Stat Med*. 2016;35(16):2741-2753. doi:10.1002/sim.6893

4. Cox DR. Regression Models and Life-Tables. *J R Stat Soc Ser B Methodol*. 1972;34(2):187-202. doi:10.1111/j.2517-6161.1972.tb00899.x

5. Simon N, Friedman J, Hastie T, Tibshirani R. Regularization Paths for Cox’s Proportional Hazards Model via Coordinate Descent. *J Stat Softw*. 2011;39(5):1-13. doi:10.18637/jss.v039.i05

6. Friedman JH, Roosen CB. An introduction to multivariate adaptive regression splines. *Stat Methods Med Res*. 1995;4(3):197-217. doi:10.1177/096228029500400303

7. Lee ET, Welty TK, Fabsitz R, et al. The Strong Heart Study. A study of cardiovascular disease in American Indians: design and methods. *Am J Epidemiol*. 1990;132(6):1141-1155. doi:10.1093/oxfordjournals.aje.a115757

8. Moon KA, Guallar E, Umans JG, et al. Association between exposure to low to moderate arsenic levels and incident cardiovascular disease. A prospective cohort study. *Ann Intern Med*. 2013;159(10):649-659. doi:10.7326/0003-4819-159-10-201311190-00719

9. Tellez-Plaza M, Guallar E, Howard BV, et al. Cadmium exposure and incident cardiovascular disease. *Epidemiol Camb Mass*. 2013;24(3):421-429. doi:10.1097/EDE.0b013e31828b0631

10. Zhao D, Domingo-Relloso A, Tellez-Plaza M, et al. High Level of Selenium Exposure in the Strong Heart Study: A Cause for Incident Cardiovascular Disease? *Antioxid Redox Signal*. 2022;37(13-15):990-997. doi:10.1089/ars.2022.0029

11. Nigra AE, Howard BV, Umans JG, et al. Urinary tungsten and incident cardiovascular disease in the Strong Heart Study: An interaction with urinary molybdenum. *Environ Res*. 2018;166:444-451. doi:10.1016/j.envres.2018.06.015

12. Pang Y, Peng RD, Jones MR, et al. Metal mixtures in urban and rural populations in the US: The Multi-Ethnic Study of Atherosclerosis and the Strong Heart Study. *Environ Res*. 2016;147:356-364. doi:10.1016/j.envres.2016.02.032

13. Scheer J, Findenig S, Goessler W, et al. Arsenic species and selected metals in human urine: validation of HPLC/ICPMS and ICPMS procedures for a long-term population-based epidemiological study. *Anal Methods Adv Methods Appl*. 2012;4(2):406-413. doi:10.1039/C2AY05638K

14. Gillum RF, Fortmann SP, Prineas RJ, Kottke TE. International diagnostic criteria for acute myocardial infarction and acute stroke. *Am Heart J*. 1984;108(1):150-158. doi:10.1016/0002-8703(84)90558-1

15. Levey AS, Stevens LA, Schmid CH, et al. A New Equation to Estimate Glomerular Filtration Rate. *Ann Intern Med*. 2009;150(9):604-612.

16. Bolt AM, Mann KK. Tungsten: an Emerging Toxicant, Alone or in Combination. *Curr Environ Health Rep*. 2016;3(4):405-415. doi:10.1007/s40572-016-0106-z

17. Kuo CC, Balakrishnan P, Gribble MO, et al. The association of arsenic exposure and arsenic metabolism with all-cause, cardiovascular and cancer mortality in the Strong Heart Study. *Environ Int*. 2022;159:107029. doi:10.1016/j.envint.2021.107029

18. Moon K, Guallar E, Navas-Acien A. Arsenic Exposure and Cardiovascular Disease:An Updated Systematic Review. *Curr Atheroscler Rep*. 2012;14(6):542-555. doi:10.1007/s11883-012-0280-x

19. Tellez-Plaza M, Navas-Acien A, Menke A, Crainiceanu CM, Pastor-Barriuso R, Guallar E. Cadmium Exposure and All-Cause and Cardiovascular Mortality in the U.S. General Population. *Environ Health Perspect*. 2012;120(7):1017-1022. doi:10.1289/ehp.1104352

20. Hall MN, Niedzwiecki M, Liu X, et al. Chronic arsenic exposure and blood glutathione and glutathione disulfide concentrations in Bangladeshi adults. *Environ Health Perspect*. 2013;121(9):1068-1074. doi:10.1289/ehp.1205727

21. Niedzwiecki MM, Hall MN, Liu X, et al. A Dose–Response Study of Arsenic Exposure and Global Methylation of Peripheral Blood Mononuclear Cell DNA in Bangladeshi Adults. *Environ Health Perspect*. 2013;121(11-12):1306-1312. doi:10.1289/ehp.1206421

22. Domingo-Relloso A, Makhani K, Riffo-Campos AL, et al. Arsenic Exposure, Blood DNA Methylation, and Cardiovascular Disease. *Circ Res*. 2022;131(2):e51-e69. doi:10.1161/CIRCRESAHA.122.320991

23. Domingo-Relloso A, Riffo-Campos AL, Haack K, et al. Cadmium, Smoking, and Human Blood DNA Methylation Profiles in Adults from the Strong Heart Study. *Environ Health Perspect*. 2020;128(6):067005. doi:10.1289/EHP6345

24. Takiguchi M, Achanzar WE, Qu W, Li G, Waalkes MP. Effects of cadmium on DNA-(Cytosine-5) methyltransferase activity and DNA methylation status during cadmium-induced cellular transformation. *Exp Cell Res*. 2003;286(2):355-365. doi:10.1016/s0014-4827(03)00062-4

25. Neumann M, Leimkühler S. Heavy metal ions inhibit molybdoenzyme activity by binding to the dithiolene moiety of molybdopterin in Escherichia coli. *FEBS J*. 2008;275(22):5678-5689. doi:10.1111/j.1742-4658.2008.06694.x

26. Wang X, Mukherjee B, Karvonen-Gutierrez CA, et al. Urinary Metal Mixtures and Longitudinal Changes in Glucose Homeostasis: The Study of Women’s Health Across the Nation (SWAN). *Environ Int*. 2020;145:106109. doi:10.1016/j.envint.2020.106109

27. Martinez-Morata I, Sobel M, Tellez-Plaza M, Navas-Acien A, Howe CG, Sanchez TR. A State-of-the-Science Review on Metal Biomarkers. *Curr Environ Health Rep*. 2023;10(3):215-249. doi:10.1007/s40572-023-00402-x

28. Martinez-Morata I, Schilling K, Glabonjat RA, et al. Association of Urinary Metals With Cardiovascular Disease Incidence and All-Cause Mortality in the Multi-Ethnic Study of Atherosclerosis (MESA). *Circulation*. 0(0). doi:10.1161/CIRCULATIONAHA.124.069414

29. Fukunaka A, Fujitani Y. Role of Zinc Homeostasis in the Pathogenesis of Diabetes and Obesity. *Int J Mol Sci*. 2018;19(2):476. doi:10.3390/ijms19020476

30. Liu B, Feng W, Wang J, et al. Association of urinary metals levels with type 2 diabetes risk in coke oven workers. *Environ Pollut Barking Essex 1987*. 2016;210:1-8. doi:10.1016/j.envpol.2015.11.046

31. Galvez-Fernandez M, Powers M, Grau-Perez M, et al. Urinary Zinc and Incident Type 2 Diabetes: Prospective Evidence From the Strong Heart Study. *Diabetes Care*. 2022;45(11):2561-2569. doi:10.2337/dc22-1152

**Tables**

| Table S1. Quantities of Interest | | |
| --- | --- | --- |
| Effect | **Scale** | **Formula** |
| Individual Metal | Multiplicative | $\frac{\lambda_{j}^{75}(t\vert\boldsymbol{M}_{-j}\boldsymbol{,C})}{\lambda_{j}^{25}(t\vert\boldsymbol{M}_{-j},C)}$ |
| Individual Metal | Additive | $S_{j}^{75}(t\vert\boldsymbol{M}_{-j}\boldsymbol{,C})-S_{j}^{25}(t\vert\boldsymbol{M}_{-j}\boldsymbol{,C})$ |
| Metal Mixture | Multiplicative | $\frac{\lambda_{1, \ldots, J}^{75, \ldots, 75}(t\boldsymbol{\vert C})}{\lambda_{1, \ldots, J}^{25, \ldots, 25}(t\boldsymbol{\vert C})}$ |
| Metal Mixture | Additive | $S_{1, \ldots, J}^{75, \ldots, 75}\left( t \vert\boldsymbol{C} \right)-S_{1, \ldots, J}^{25, \ldots, 25}(t\boldsymbol{\vert C})$ |
| Interaction | Multiplicative | $\frac{\lambda_{j,j^{'}}^{75, 75}(t\vert\boldsymbol{M}_{-\{j,j^{'}\}}\boldsymbol{,C})\times\lambda_{j,j^{'}}^{25,25}(t\vert\boldsymbol{M}_{-\{j,j^{'}\}}\boldsymbol{,C})}{\lambda_{j,j^{'}}^{25, 75}(t\vert\boldsymbol{M}_{-\{j,j^{'}\}}\boldsymbol{,C})\times\lambda_{j,j^{'}}^{75,25}(t\vert\boldsymbol{M}_{-\{j,j^{'}\}}\boldsymbol{,C})}$ |
| $\boldsymbol{\lambda}_{\boldsymbol{j\subset}\left\{ \boldsymbol{1,\ldots,J} \right\}}^{\boldsymbol{q}_{\boldsymbol{1}}\boldsymbol{,\ldots,}\boldsymbol{q}_{\left\vert\boldsymbol{j} \right\vert}}\boldsymbol{(t\vert}\boldsymbol{M}_{\boldsymbol{-j}}\boldsymbol{, C)}$ and $\boldsymbol{S}_{\boldsymbol{j\subset}\left\{ \boldsymbol{1,\ldots,J} \right\}}^{\boldsymbol{q}_{\boldsymbol{1}}\boldsymbol{,\ldots,}\boldsymbol{q}_{\left\vert\boldsymbol{j} \right\vert}}\boldsymbol{(t\vert}\boldsymbol{M}_{\boldsymbol{-j}}\boldsymbol{C)}$ denote the hazards and survival probability, respectively, at a specified time *t* when exposed to environmental mixture components who’s indices are in $\boldsymbol{j}$ at their respective $\boldsymbol{q}_{\boldsymbol{1}}^{\boldsymbol{th}}\boldsymbol{, \ldots,}\boldsymbol{q}_{\boldsymbol{\vert j\vert}}^{\boldsymbol{th}}$ percentiles, conditional on all other metals $\boldsymbol{M}_{\boldsymbol{-j}}\boldsymbol{,}$ and all covariates $\boldsymbol{C}$. | | |

| Table S2. Summary of method’s chosen hyperparameters and run time* | | | |
| --- | --- | --- | --- |
| Method | Hyper- Parameter | Grid of Values Considered | Median Run Time (IQR) |
| Cox Proportional Hazards Model | none |  | 6.8 minutes (6.6, 7.1)  11.2 minutes (10.0, 11.8)** |
| Proportional Hazards Model with Penalized Splines | none |  | 6.7 hours (6.1, 7.2) |
| Cox Proportional Hazards Model with Elastic Net | $\omega$ | {0.0, 0.2, …, 1.0} | 33.5 minutes (32.6, 34.7)  7.2 hours (6.9, 7.5)** |
|  | $\kappa$ | Pathwise solution |  |
| Multivariate Adaptive Regression Splines | $P$ | {2, 4, …, 30} | 3.9 hours (3.7, 4.3)*** |
|  | $D$ | {1,2} |  |
| Bayesian Additive Regression Tree | $P$ | 50 | 47.0 minutes (46.9, 47.6) |
|  | $k$ | 2 |  |
|  | $a$ | 0.95 |  |
|  | $b$ | 2 |  |
| *Run time for simulated data with sample size of $\boldsymbol{n=3000}$ and 500 bootstrap samples for frequentist methods  **Average run time for the model with interaction terms  ***Cross-validation for MARS model done in parallel over 4 cores | | | |

| Table S3. Baseline characteristics of Strong Heart Study participants (n=2728) | |
| --- | --- |
| Mean age (SE), years | 56.1 (0.2) |
| Female, % | 59.2 |
| Education, % |  |
| No high school | 17.2 |
| Some high school | 23.4 |
| Completed high school | 59.4 |
| Mean BMI (SE), kg/m^2^ | 30.4 (0.1) |
| Smoking, % |  |
| Never | 29.3 |
| Former | 32.9 |
| Current | 37.8 |
| Mean Arsenobetaine (SE), µg/L | -0.1 (0.02) |
| Mean eGFR (SE), mL/min/1.73 m^2^ | 97.5 (0.3) |
| Median Creatine Adjusted Metal Concentrations (IQR), µg/g |  |
| Arsenic^a^ | 8.42 (5.15, 14.32) |
| Cadmium | 0.96 (0.62, 1.51) |
| Molybdenum | 29.36 (20.46, 41.39) |
| Selenium | 48.99 (36.73, 67.35) |
| Tungsten | 0.12 (0.06, 0.23) |
| Zinc | 561.30 (389.50, 805.35) |
| ^a^Arsenic exposure is measured as the sum of inorganic and methylated arsenic species  Abbreviations: SE, standard error; eGFR, estimated glomerular filtration rate (a quantification of kidney function) | |

**Figures**

***
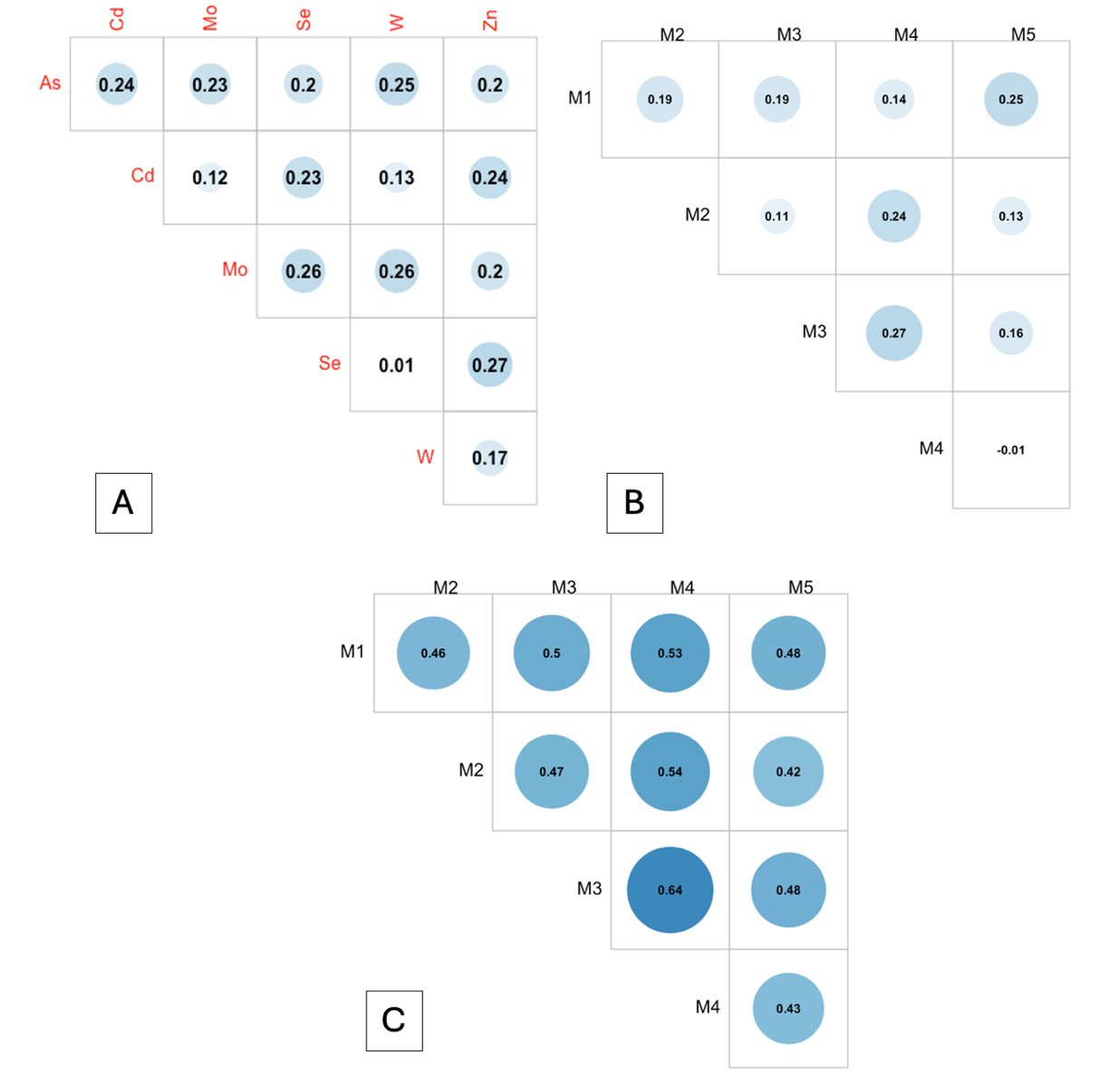
***

***Figure S1.*** *Correlation matrices for various datasets.* *Panel A are the observed correlations across the log transformed urine metal concentrations at baseline in the Strong Heart Study. Panels B and C are examples of correlation matrices across metal mixture components for simulated mixtures. Panel B is for scenarios where exposures are low to moderately correlated and Panel C is for the scenario where exposures are highly correlated.*


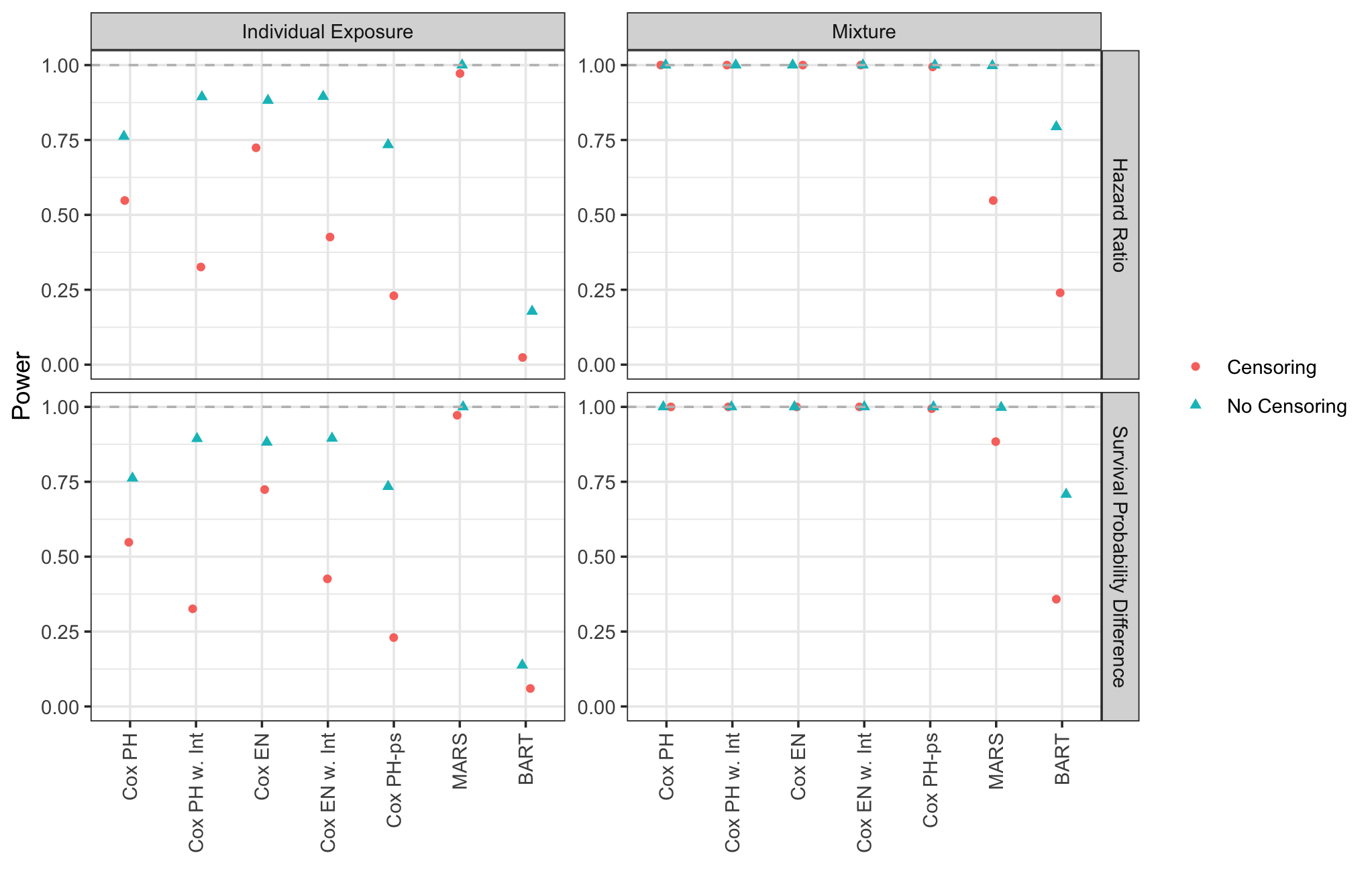


***Figure S2.*** *Probability of detecting a significant effect (power) across methods under ~30% censoring compared to no censoring.*

*
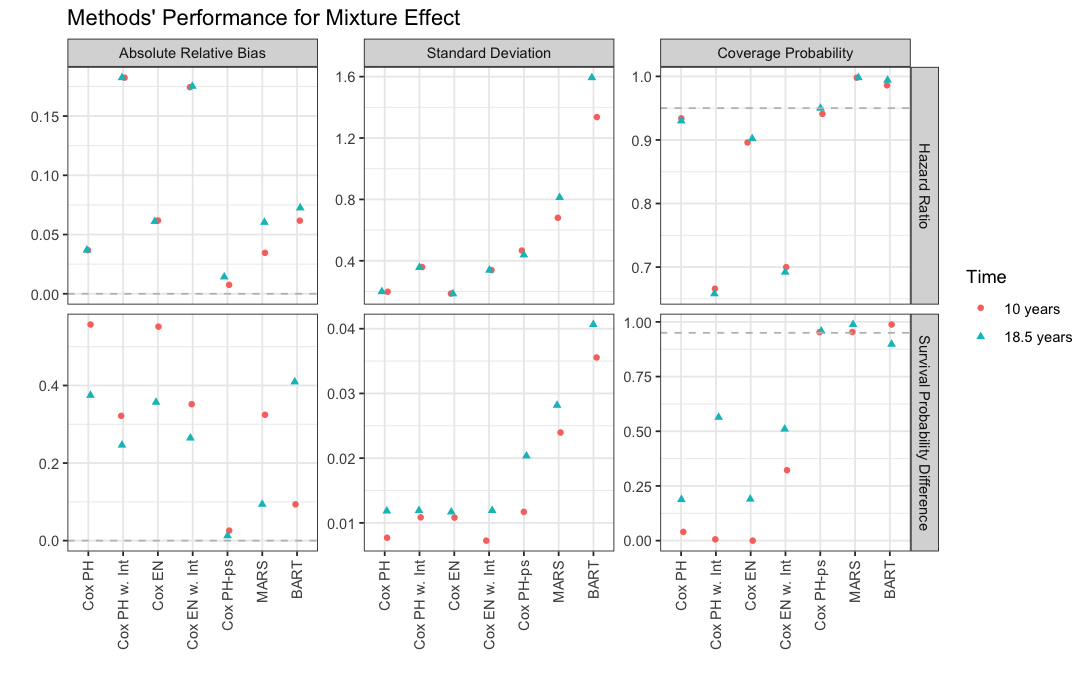
*

**Figure S3.** Comparison of methods’ estimates of the hazard ratio and survival probability difference for an interquartile range change in each component of a mixture for the base-case at two time points: 10 years and the 80^th^ percentile of observed time, 18.5 years.


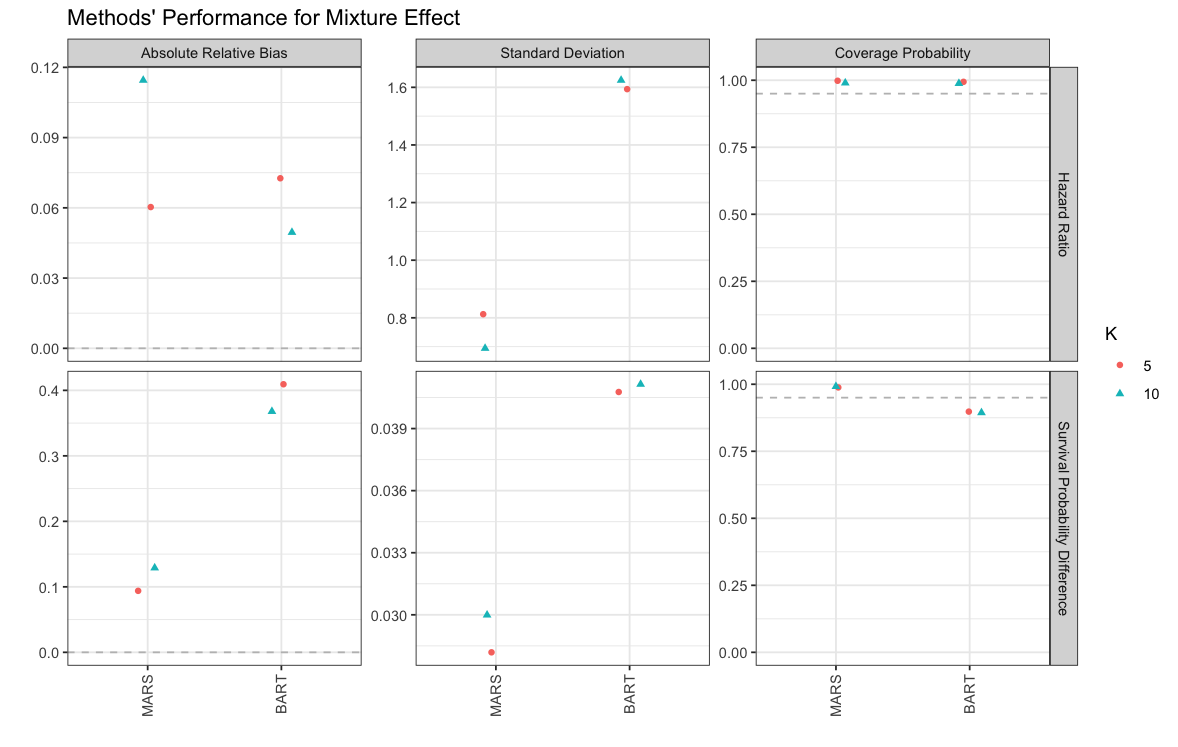


**Figure S4.** Comparison of the discrete-time survival analysis methods’ estimates of the hazard ratio and survival probability difference for an interquartile range change in each component of a mixture for the base-case when using different number of bins (K) when running the model.


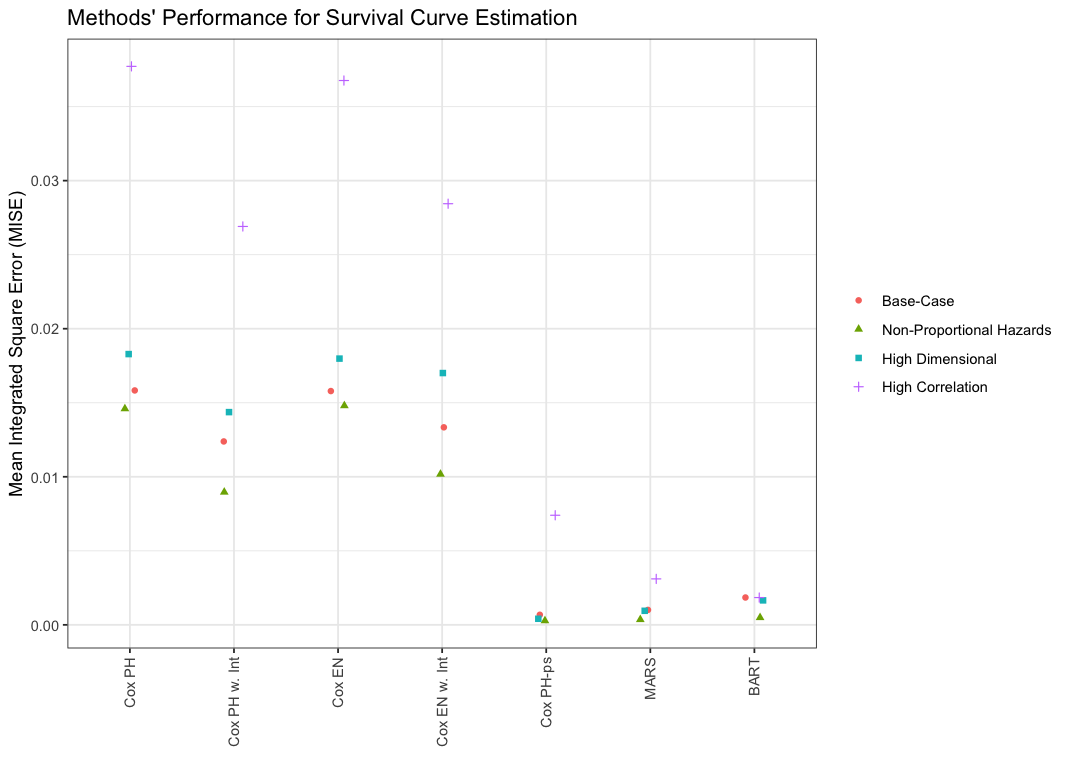


**Figure S5.** Mean integrated squared error (MISE) for the exposure-response curves for a single metal (labeled as “Metal 3” in Figure 4 from the main manuscript) under various simulation scenarios.

*
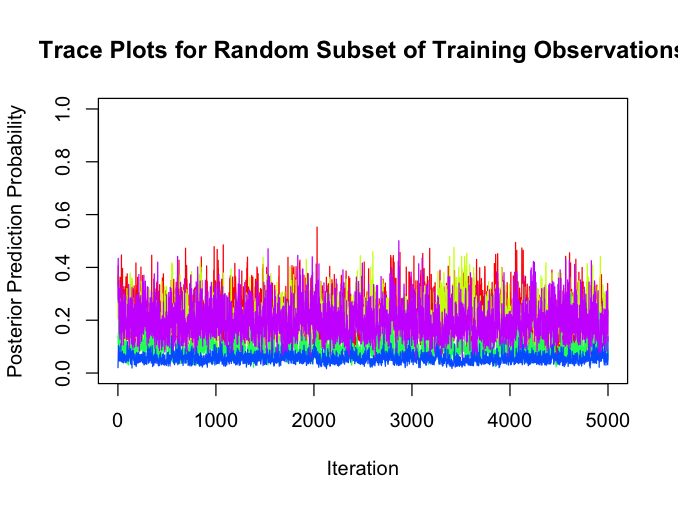
*
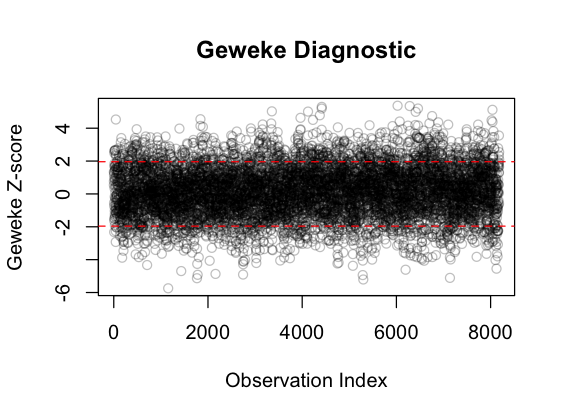
*
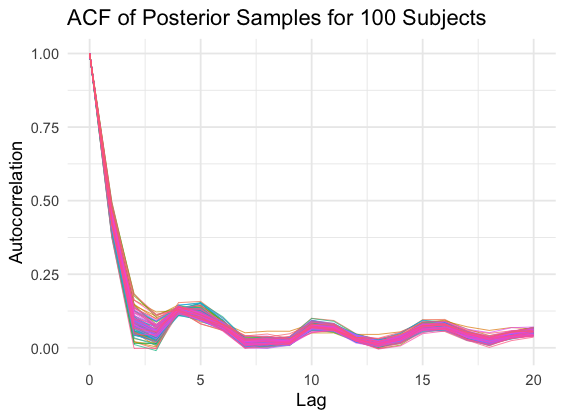
*

**Figure S6.** Diagnostic plots for convergence assessment of the BART model from Strong Heart Study data.

**Figure S7.** Estimated association of mixture with cardiovascular disease in the Strong Heart Study, quantified as the hazard ratio (HR) and survival probability difference (SPD) for an interquartile range change in arsenic (As), cadmium (Cd), molybdenum (Mo), selenium (Se), tungsten (W), and zinc (Zn), and estimated using different modeling methods; results are shown at two time points, 10 years and 80^th^ percentile of follow-up (18.4 years).


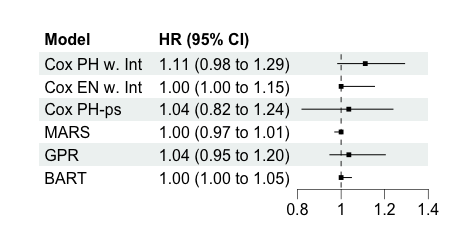


**Figure S8.** Estimated interaction effect between Se and W on incident cardiovascular disease for the Strong Heart Study, quantified as the multiplicative interaction. Estimates shown at the 80^th^ percentile of follow-up (18.4 years) and only for the models which allow for interaction estimations.
